# SOBIR1 integrates pathogen recognition and hormonal metabolism in *Nicotiana tabacum*

**DOI:** 10.1093/plphys/kiag707

**Published:** 2026-09-23

**Authors:** Adam Janotík, Martina Zapletalová, Karim Bouhidel, Jérôme Fromentin, Markéta Lišková, David Novák, Ivan Petřík, Ondřej Novák, Jaromir Mikulik, Jan Strnad, Ján Šmeringai, Markéta Pernisová, Nathalie Leborgne-Castel, Jan Lochman

**Affiliations:** Department of Biochemistry, Faculty of Science, Masaryk University, Kotlářská 2, Brno 61137, Czech Republic; Université Bourgogne Europe, Institut Agro, INRAE, UMR Agroécologie, 17 rue Sully, 21065 Dijon cedex, France; Department of Biochemistry, Faculty of Science, Masaryk University, Kotlářská 2, Brno 61137, Czech Republic; Université Bourgogne Europe, Institut Agro, INRAE, UMR Agroécologie, 17 rue Sully, 21065 Dijon cedex, France; Université Bourgogne Europe, Institut Agro, INRAE, UMR Agroécologie, 17 rue Sully, 21065 Dijon cedex, France; Department of Biochemistry, Faculty of Science, Masaryk University, Kotlářská 2, Brno 61137, Czech Republic; Department of Biochemistry, Faculty of Science, Masaryk University, Kotlářská 2, Brno 61137, Czech Republic; Laboratory of Growth Regulators and Department of Chemical Biology and Genetics, Centre of the Region Haná for Biotechnological and Agricultural Research, Faculty of Science, Palacký University and Institute of Experimental Botany ASCR, Šlechtitelů 27, Olomouc 78371, Czech Republic; Laboratory of Growth Regulators and Department of Chemical Biology and Genetics, Centre of the Region Haná for Biotechnological and Agricultural Research, Faculty of Science, Palacký University and Institute of Experimental Botany ASCR, Šlechtitelů 27, Olomouc 78371, Czech Republic; Laboratory of Growth Regulators and Department of Chemical Biology and Genetics, Centre of the Region Haná for Biotechnological and Agricultural Research, Faculty of Science, Palacký University and Institute of Experimental Botany ASCR, Šlechtitelů 27, Olomouc 78371, Czech Republic; Laboratory of Growth Regulators and Department of Chemical Biology and Genetics, Centre of the Region Haná for Biotechnological and Agricultural Research, Faculty of Science, Palacký University and Institute of Experimental Botany ASCR, Šlechtitelů 27, Olomouc 78371, Czech Republic; National Center for Biomolecular Research, Faculty of Science, Masaryk University, Kotlářská 2, Brno 61137, Czech Republic; Mendel Centre for Plant Genomics and Proteomics, Central European Institute of Technology, Masaryk University, Kamenice 5, 62500 Brno, Czechia; National Center for Biomolecular Research, Faculty of Science, Masaryk University, Kotlářská 2, Brno 61137, Czech Republic; Mendel Centre for Plant Genomics and Proteomics, Central European Institute of Technology, Masaryk University, Kamenice 5, 62500 Brno, Czechia; Université Bourgogne Europe, Institut Agro, INRAE, UMR Agroécologie, 17 rue Sully, 21065 Dijon cedex, France; Department of Biochemistry, Faculty of Science, Masaryk University, Kotlářská 2, Brno 61137, Czech Republic

## Abstract

The receptor-like kinase SOBIR1 (SUPPRESSOR OF BIR1) is a core component of immune receptor complexes mediating plant responses to extracellular immunogenic signals. While its role in pattern-triggered immunity is well defined in model species, its broader physiological functions remain largely unexplored. Here, we generated CRISPR–Cas9-derived double knockouts of 2 homeologous *SOBIR1* genes in allotetraploid *Nicotiana tabacum*. The resulting *Nt sobir1* double mutants failed to trigger elicitor-induced cell death in response to elicitins from *Phytophthora* and *Pythium* spp. but retained an attenuated early reactive oxygen species (ROS) response while lacking sustained ROS accumulation, showing that the transient and sustained ROS outputs differ in their dependence on SOBIR1. Functionally, the mutants showed compromised resistance to *Phytophthora parasitica* but unaltered responses to *Botrytis cinerea* and *Pseudomonas syringae*, pointing to pathogen-specific SOBIR1 roles. Beyond immunity, SOBIR1 appears to interface with developmental signaling modules involving CLAVATA2 and ERECTA–YODA, implicating it in phytohormone signaling, meristem regulation, and cell proliferation. Notably, *Nt sobir1* mutants displayed increased hypocotyl cell wall stiffness despite transcriptional upregulation of wall-loosening genes, suggesting compensatory cell wall remodeling and perturbed integrity signaling. Transcriptome and phytohormone profiling revealed tissue-specific expression changes and altered levels of cytokinins, salicylic acid, jasmonic acid, and their conjugates. These findings position SOBIR1 as a multifunctional regulatory hub that links immune perception with phytohormone dynamics and cell wall mechanics in *N. tabacum*, expanding our understanding of co-receptor signaling plasticity across plant lineages.

## Introduction

Plants possess a multilayered innate immune system, with the first layer relying on recognition of extracellular immunogenic patterns (ExIPs), including microbial-associated molecular patterns, damage-associated molecular patterns, and phytocytokines, which are plant endogenous peptides released into the apoplast (Boller and Felix 2009; Gust et al. 2017; van der Burgh and Joosten 2019). Pattern perception is mediated by diverse plasma membrane (PM) pattern recognition receptors (PRRs) that confer durable resistance in model and crop species. PRRs are classified by the presence or absence of an intracellular kinase domain into receptor-like kinases (RLKs) and receptor-like proteins (RLPs) (Macho and Zipfel 2014). RLPs are single-pass transmembrane proteins with ligand-binding extracellular domains and short cytoplasmic tails lacking kinase activity, typically forming constitutive complexes with RLKs (Zipfel 2014). In various plant species, perception of ExIPs activates downstream receptor-like cytoplasmic kinases (Hailemariam et al. 2024), mitogen-activated protein kinase (MAPK) cascades, or the production of reactive oxygen species (ROS), resulting in transcriptional reprogramming and extracellularly triggered immunity (ExTI) (Birkenbihl et al. 2017). This signaling depends not solely on the PRRs but also on the associated immune signaling complexes. Many leucine-rich repeat (LRR)-RLKs signal via complexes with BRI1-ASSOCIATED KINASE 1/SOMATIC EMBRYOGENESIS RECEPTOR KINASE 3 (BAK1/SERK3) (Chinchilla et al. 2009), while numerous LRR-RLPs consistently associate with SUPPRESSOR OF BIR1-1 (SOBIR1). These LRR-RLP/SOBIR1 complexes have been described as bipartite receptors, with the RLP providing the ligand specificity and SOBIR1 providing the kinase domain for intracellular signaling (Liebrand et al. 2014). Upon recognition of the ligand by the LRR-RLP, SOBIR1 is *trans*-phosphorylated by the RLK BAK1/SERK3 following its autophosphorylation via an intermolecular mechanism (Wei et al. 2022). These tripartite signaling units are key players in ExTI and play critical roles in modulating plant-pathogen interactions relevant to horticultural crop health (van der Burgh et al. 2019).

To date, SOBIR1 has been described interacting in complexes with diverse LRR-RLPs (Wulff et al. 2004; Wang et al. 2010; de Jonge et al. 2012; Liebrand et al. 2013; Du et al. 2015; Hegenauer et al. 2016; Albert et al. 2019; Huang et al. 2020; Sussholz et al. 2020; Zhang et al. 2021; Chen et al. 2022, 2023; Fan et al. 2022), particularly relevant in solanaceous horticultural crops (such as *Solanum lycopersicum*), where diseases like leaf mold, caused by *Cladosporium fulvum*, and late blight, caused by *Phytophthora infestans*, present persistent threats to yield and quality. However, the role of SOBIR1, also known as the LRR-RLK EVERSHED (EVR) (Leslie et al. 2010), is multifaceted, functioning in signal transduction pathways that regulate not only immune responses but also plant growth and development processes, illustrating its dual role in immunity and morphogenesis, which has implications for crop architecture and productivity (Liebrand et al. 2013). Moreover, research has revealed that in tomatoes, SOBIR1 interacts with the LRR-RLP CLAVATA2 (CLV2), which regulates various pathways related to development and immune signaling (Liebrand et al. 2013).

SOBIR1 has also been linked to the detection of elicitins in *Solanum microdontum* (Du et al. 2015; Domazakis et al. 2018) and *Nicotiana benthamiana* (Chen et al. 2023). Elicitins are small, structurally conserved proteins secreted by oomycetes in the *Phytophthora* and *Pythium* genera, known to trigger hypersensitive response (HR) and systemic acquired resistance (SAR) in plants (Colas et al. 2001). A potential receptor for elicitins, the LRR-RLP termed ELICITIN RESPONSE (ELR), was first identified in the wild potato species *S. microdontum* (Du et al. 2015). More recently, the homologous LRR-RLP receptor gene, named *RESPONSIVE TO ELICITINS* (*REL*), was discovered in *N. benthamiana*. In *Nb rel* knockout mutants failed to trigger HR upon elicitins exposure and showed enhanced susceptibility to *P. infestans* and *Phytophthora parasitica* (Chen et al. 2023). Both ELR and REL receptors have been shown to form bipartite complexes with SOBIR1 on the PM, and following elicitin activation, the LRR-RLK SERK3/BAK1 (a general co-receptor of activated RLP/SOBIR1) was recruited to this complex (Domazakis et al. 2018; Chen et al. 2023).

In this study, CRISPR–Cas9-generated *Nt* sobir1 double mutants and derived cell suspensions of *Nicotiana tabacum* cv. Xanthi were used to investigate NtSOBIR1 function in elicitin-triggered immunity. The results show that SOBIR1 is required for sustained β-cryptogein-induced ROS accumulation and HR-like cell death. Nevertheless, the mutants retained an attenuated transient ROS response, indicating that the transient and sustained signaling outputs differ in their degree of dependence on NtSOBIR1.

## Results

### Identification of 2 SOBIR1 genes of *N. tabacum* and their place in the phylogenetic tree of Solanaceae

We identified 2 homeologs *SOBIR1* genes in the tobacco (*N. tabacum*) genome, designated *NtSOBIR1-1* and *NtSOBIR1-2*, both showing strong homology to the *Arabidopsis thaliana SOBIR1* gene. *N. tabacum* is an allotetraploid species derived from *Nicotiana sylvestris* and *Nicotiana tomentosiformis* (Sierro et al. 2024), and each *NtSOBIR1* gene clusters closely with its respective progenitor homolog (Fig. 1a). The predicted *Nt*SOBIR1 proteins share ∼62% amino acid identity with *At*SOBIR1 and 95.6% identity with each other (GenBank: XP_016441673.1 and NP_001312335.1). To investigate the evolutionary relationships of SOBIR1 proteins within the *Solanaceae*, we constructed a phylogenetic tree including 160 putative SOBIR1 homologs from 85 species (Fig. S1). In the *N. benthamiana* reference genome (assembly ASM3437652v1) (Ko et al. 2024), we identified 2 genes homologous to *AtSOBIR1*, designated *NbSOBIR1-1* and *NbSOBIR1-2* (Figs. 1a, S2a and S3). *NbSOBIR1-1*, previously described by Liebrand et al. (2013) and Peng et al. (2015) as *NbSOBIR1*, is located on chromosome 11 and consists of a single open reading frame (ORF; Fig. S2a). RNA-seq data indicate that *NbSOBIR1-1* is expressed in both seedlings and mature plants (Fig. S2b). *NbSOBIR1-2* is located on chromosome 9 and contains a single ORF that is 96.7% identical to that of *NbSOBIR1-1* (Figs. S2a and S3). Although transcripts derived from *NbSOBIR1-2* were detected, their abundance was substantially lower (Fig. S2b). In addition to these 2 functional loci, we identified in *N. benthamiana* 2 additional genomic regions with homology to *AtSOBIR1*. The first, designated *NbSOBIR1-PS1*, is located on chromosome 3 (Fig. S2a) and corresponds to the *NbSOBIR1*-like sequence previously reported by Liebrand et al. (2013) and Huang et al. (2020). The sequence retrieved from the reference genome differs slightly from the published sequence (Fig. S4) and contains 2 insertions of 23 and 234 bp. The larger insertion shares 92.6% nucleotide identity with Nt3, a short interspersed nuclear element previously described in *N. tabacum* (Yoshioka et al. 1993). *NbSOBIR1-PS1* shares 78.9% nucleotide identity with *NbSOBIR1-1* (Fig. S5) but lacks an intact ORF due to the Nt3 insertion, multiple frameshift mutations, and premature stop codons (Fig. S2a). Consistent with this, transcripts derived from *NbSOBIR1-PS1* were detected at extremely low levels, if at all, in RNA-seq datasets (Fig. S2b). The second locus, designated *NbSOBIR1-PS2*, is located on chromosome 13 and corresponds to a predicted cDNA (ID NbS00006092g015.1) reported by Peng et al. (2015) as *NbSOBIR1*-like. This cDNA is predicted to encode a 480-amino acid protein, substantially shorter than the 634-amino acid *Nb*SOBIR1-1 protein. Alignment of genomic and cDNA sequences revealed that the predicted transcript would require splicing of 2 hypothetical introns (Fig. S6) and uncovered 3 nucleotide discrepancies within exon 1, including a single-nucleotide deletion in the genomic sequence. If this splicing pattern were correct, the resulting transcript would encode only a 129-amino acid protein (first ORF in Fig. S2a). RNA-seq reads mapping to the *NbSOBIR1-PS2* locus were rare and did not support the predicted splicing pattern (Fig. S2b). Together, these findings indicate that the loci on chromosomes 3 and 13 represent *SOBIR1* pseudogenes. Consequently, their predicted translation products were excluded from the phylogenetic analysis. This phylogenetic reconstruction revealed 2 intron-less clades, designated Clade 1 and Clade 2 (Fig. S1). Most diploid species possess one gene from each clade, whereas *Nicotiana* species contain only Clade 1 members.

**Figure 1:**
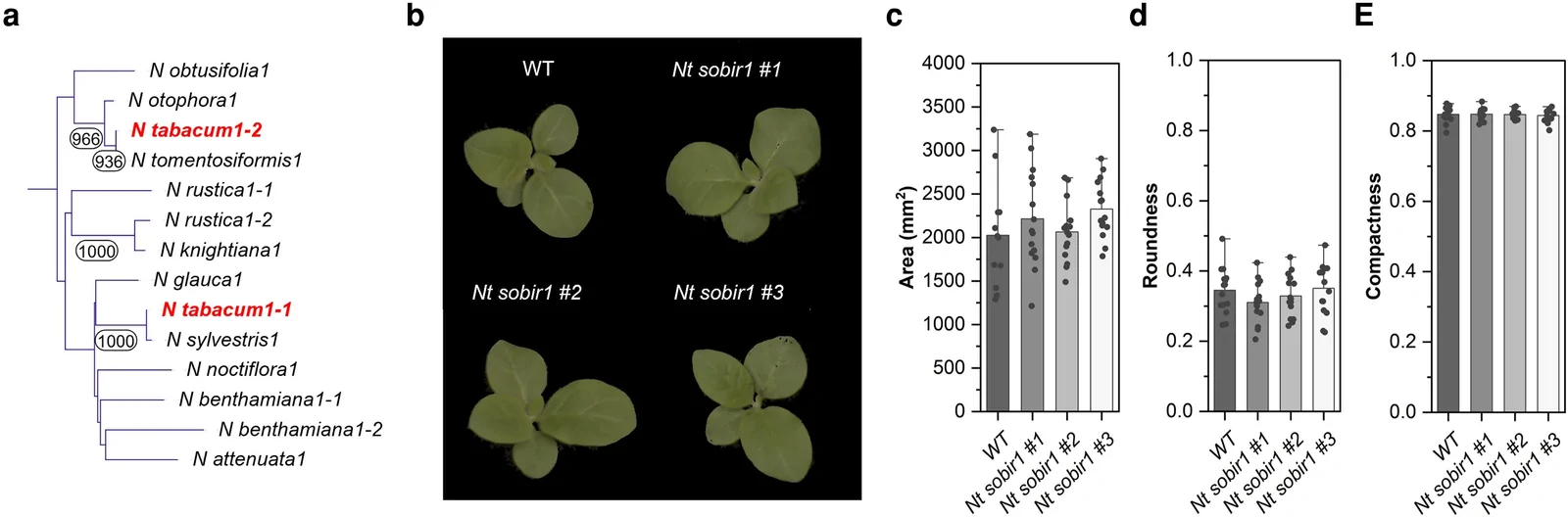
Phylogenetic relationships and phenotypic characterization of *Nt sobir1* mutants from *N. tabacum*. a) Phylogenetic relationships of *SOBIR1* homologs from 11 *Nicotiana* species. The tree is a subtree excerpted from a broader phylogeny of Solanaceae SOBIR1 homologs (see Fig. S1), constructed using FastME with 1,000 bootstrap replications. Tobacco homeologs are highlighted in red, and bootstrap values above 900 are shown below the corresponding branches. b) Representative images of wild-type (WT) and Nt sobir1 double mutant lines (*Nt sobir1* #1, #2, and #3) of *Nicotiana tabacum* cv. Xanthi at 21 days post-sowing. c to e) Phenotypic analysis of rosette area c), roundness d), and compactness e). No significant differences were detected (1-way ANOVA; *n* = 20 plants).

### Generation and characterization of CRISPR–Cas9-derived Nt sobir1 double mutant tobacco lines

In *N. tabacum*, the 2 *NtSOBIR1* homeologous genes are expressed at similar levels in the leaves of cultivar Xanthi (Fig. S7), suggesting redundant or overlapping functions. We therefore designed a CRISPR–Cas9 strategy to simultaneously inactivate both *NtSOBIR1* genes to investigate their roles in development, physiology, and defense.

Three distinct F_2_ homozygous double-knockout lines for *Nt sobir1-1* and *sobir1-2* were generated, each carrying distinct mutations in the *NtSOBIR1-1* and *NtSOBIR1-2* genes. For clarity, these lines are hereafter referred to as: *Nt sobir1 #1*, *Nt sobir1 #2*, and *Nt sobir1 #3* (Fig. S7). First, to assess the effects of these mutations on their expression, *NtSOBIR1* transcript levels in the F_2_ progeny of the 3 double mutant lines were quantified using RT-qPCR. The results confirmed a reduction in *NtSOBIR1* transcript levels, and sequencing of RT-qPCR products confirmed the presence of mutations in corresponding transcripts, leading to premature stop codons, generating truncated proteins (Fig. S7). Finally, phenotypic characterization of these 3 F_2_  *Nt* sobir1 double mutant lines was performed using top-view RGB imaging with the PlantScreen system (Šmeringai et al. 2023). No statistically significant differences were observed between the 3 distinct double mutant lines and wild-type (WT) controls in general rosette morphology, as defined by projected rosette area, roundness, and compactness (Fig. 1b to e).

### CRISPR–Cas9 mediated inactivation of SOBIR1 induces tissue-specific transcriptional reprogramming in leaves and roots

Although SOBIR1 is primarily recognized for its role in innate immunity, accumulating evidence indicates that it also participates in diverse developmental and cellular processes, including membrane trafficking during abscission (Leslie et al. 2010), regulation of xylem fiber differentiation (Milhinhos et al. 2019), and endosomal or Golgi-associated trafficking under stress conditions (Liebrand et al. 2014). To investigate the broader role of SOBIR1 in tobacco, we performed RNA sequencing on leaves and roots of WT *N. tabacum* plants and 2 independent double mutant lines, *Nt sobir1 #1* and *#2*. Sequencing reads (Table S1) were mapped to the *N. tabacum* reference genome (Ntab-TN90, NCBI), achieving an average mapping efficiency of ∼77%. Differential expression analysis was conducted using a threshold of *P* < 0.05 and an absolute fold change ≥2. Compared with WT plants, 2,087 and 3,580 genes were differentially expressed in leaves of *Nt sobir1 #1* and *#2*, respectively, whereas 4,677 and 3,982 genes were differentially expressed in roots of the corresponding mutant lines (Table S2). To identify NtSOBIR1-dependent transcriptional changes, we focused on differentially expressed genes (DEGs) in both double mutant lines, yielding 1,214 shared DEGs in leaves and 1,148 shared DEGs in roots (Table S2, Fig. S8). Notably, the overlap between leaf- and root-specific DEGs was minimal, indicating that loss of NtSOBIR1 triggers largely distinct transcriptional programs in aerial and below-ground tissues (Fig. S8). To characterize the biological processes underlying these tissue-specific responses, Gene Ontology enrichment analysis was subsequently performed using STRING in Cytoscape (Shannon et al. 2003; Szklarczyk et al. 2017), with a focus on biological process categories, together with analysis of hormonal and physiological responses.

### SOBIR1 inactivation alters cell wall organization and mechanical properties in leaves

In leaves, the most significantly enriched biological processes among DEGs were cell cycle regulation (including mitotic cell cycle transitions and cyclin-dependent protein kinase activity) and plant-type cell wall organization (Fig. 2a, Table S3). Among cell wall-related genes, both mutants showed increased expression of cell wall-loosening enzymes, including endoglucanases, pectate lyases, and α-expansins (Fig. 2b), which are typically associated with wall extension under acidic pH conditions. To assess whether these transcriptional changes were reflected in altered mechanical properties, we measured hypocotyl cell wall stiffness using atomic force microscopy (AFM). This technique applies precisely controlled forces to the cell surface, enabling calculation of the Young's modulus (*E*), a quantitative measure of resistance to elastic deformation. In plant cell walls, *E* directly reflects stiffness when higher values indicate more rigid walls, while lower values denote greater elasticity. In minimally deformable tissues, the force–indentation relationship allows calculation of the apparent *E*, which integrates both wall mechanics and deformation rate, providing a robust microscale measure of stiffness (Peaucelle 2014). Measurements of apparent *E* in the hypocotyl elongation zone of 14-d-old seedlings revealed significant differences between WT and *Nt sobir1 #1* and *#2* double mutant plants (*P* < 0.05, Fig. 2c). Contrary to expectations based on the up-regulation of wall-loosening genes, NtSOBIR1 knockout mutant resulted in increased cell wall stiffness, indicating that loss of NtSOBIR1 leads to complex remodeling of wall mechanics rather than simple wall loosening.

**Figure 2:**
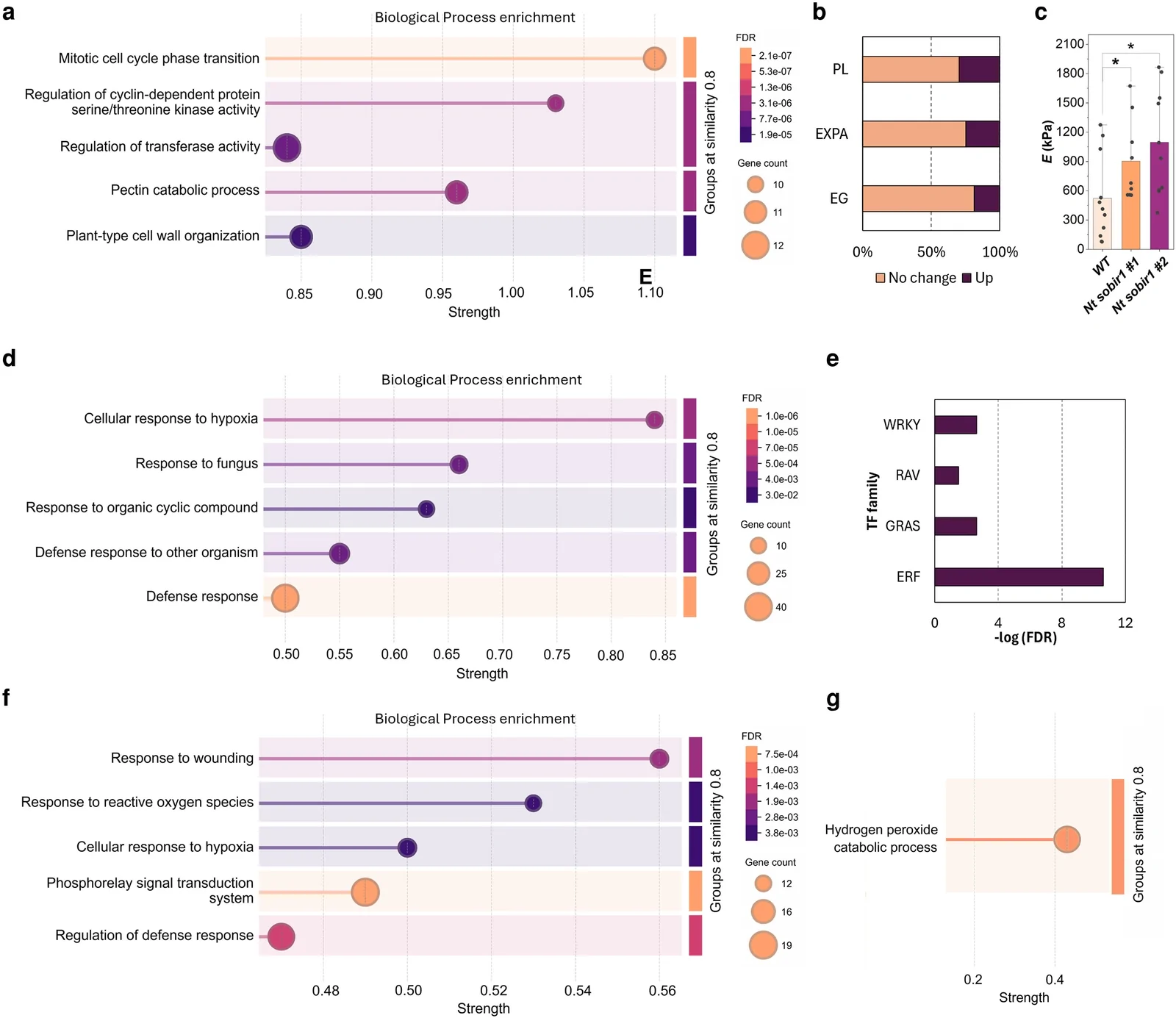
Development and cell growth processes linked to the RLK Nt SOBIR1 in leaves. a) STRING Gene Ontology (GO) biological process enrichment dot plot of genes commonly upregulated (fold change > 2, *P*adj < 0.05) in leaves of both *Nt sobir1* #1 and #2 double mutants compared with WT. GO term descriptions are shown on the *y*-axis, enrichment strength on the *x*-axis, dot size indicates the observed gene count, and color represents the false discovery rate (FDR), as defined by STRING. b) Proportion of significantly induced cell wall–modifying enzyme genes (fold change > 2, *P*_adj_ < 0.05). c) AFM analysis showing increased cell wall stiffness in hypocotyls of *Nt sobir1* #1 and #2 double mutants compared with WT. Young's modulus e) was calculated from at least 8 measurements per genotype (*n* ≥ 8); significance was determined by 1-way ANOVA (*P* < 0.05). d) STRING GO Biological Process enrichment dot plot of genes commonly downregulated (fold change > 2, *P*_adj_ < 0.05) in both *Nt sobir1* #1 and #2 double mutants compared with WT. GO term descriptions are shown on the *y*-axis, enrichment strength on the *x*-axis, dot size indicates the observed gene count, and color represents the FDR. e) Enrichment of TF families in *Nt sobir1* mutants identified by chi-squared analysis. f, g) STRING Gene Ontology (GO) biological process enrichment dot plot of genes commonly upregulated f) or downregulated g) (fold change > 2, *P*_adj_ < 0.05) in roots of both *Nt sobir1* #1 and #2 double mutants compared with WT. GO term descriptions are shown on the *y*-axis, enrichment strength on the *x*-axis, dot size indicates the observed gene count, and color represents the FDR, as defined by STRING.

### SOBIR1 inactivation suppresses MAPK–SA immune signaling and promotes jasmonate accumulation in leaves

Downregulated DEGs in leaves were significantly enriched in defense response (Fig. 2d, Table S3). Notably, several key MAPKs implicated in plant immune responses, including MPK3, MMK2, WIPK, and YODA (Pitzschke et al. 2009), were downregulated. In addition, multiple transcription factors (TFs) regulating biotic and abiotic stress responses, including ERFs, RAV factors, WRKY, and GRAS TFs, were affected (Feng et al. 2020; Wu et al. 2022; Javed and Gao 2023; Fig. 2e).

This transcriptional repression coincided with reduced salicylic acid (SA) levels and increased accumulation of *cis*-OPDA and jasmonic acid (JA) (Fig. 3a and b), indicating a shift toward JA-dominated phytohormone signaling in leaves of *Nt sobir1* double mutant plants. Notably, a subset of defense-related genes, including *PR1B*, *PR1C*, thaumatin-like proteins, and chitinase-like proteins (Ali et al. 2018; de Jesús-Pires et al. 2020; Han et al. 2023), remained up-regulated. This selective induction correlated with the increased expression of several members of the Dof TF family, which have been implicated in the regulation of pathogenesis-related and antimicrobial genes and in integrating phytohormone signals and stress responses in plants (Zou and Sun 2023). Together, these results indicate that loss of NtSOBIR1 in leaves attenuates MAPK–SA immune signaling, promotes jasmonate accumulation, and selectively activates antimicrobial defense outputs.

**Figure 3:**
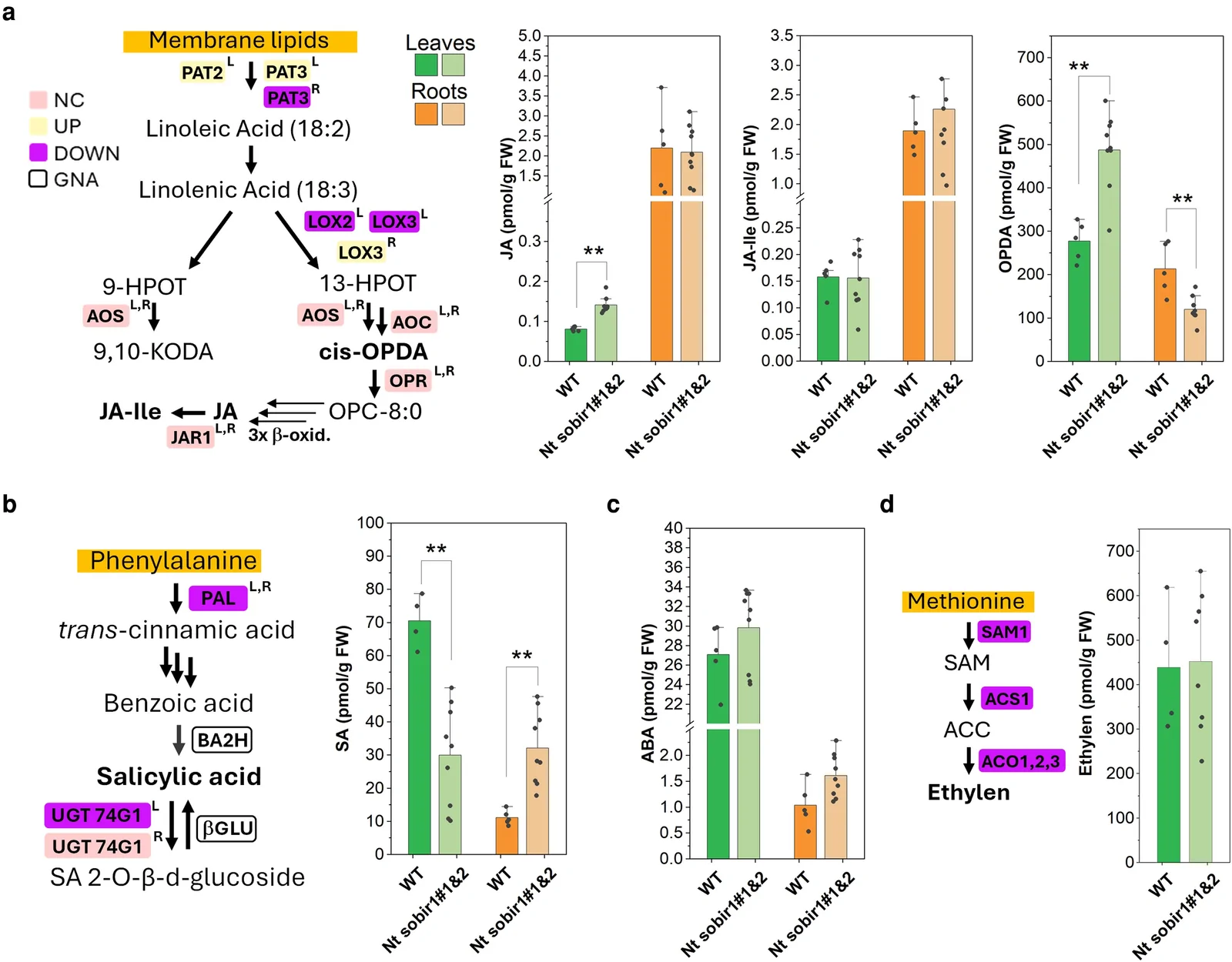
Hormonal signaling pathways are impaired in both *Nt sobir1* double mutants. The adjacent diagrams summarize major biosynthetic pathways and associated genes. Gene boxes are color-coded to reflect transcriptomic changes of key biosynthetic enzymes (UP-upregulated, DOWN-downregulated, NC-not changed and GNA-gene not annotated). Uppercase in gene boxes indicates changes in leaves (L) and roots (R). a to c) Quantification of key stress-related phytohormones JA, JA isoleucine and its precursor 12-oxo-phytodienoic acid (OPDA), SA and abscisic acid in leaves, and roots of WT and *Nt sobir1* mutant lines (#1 and #2) measured by LC-MS/MS (*n* = 5). d) Ethylene accumulation in leaves was measured by gas chromatography (*n* = 4). Data are presented as means ± standard deviation (Sd), asterisks indicate statistically significant differences in *Nt sobir1* mutant lines compared with WT (*P* < 0.01 (**); Student's *t*-test).

### SOBIR1 mutation modulates cytokinin and auxin metabolism specifically in leaves

RNA-seq analysis further revealed that both *Nt* sobir1 double mutants (*Nt sobir1 #1* and *#2*) exhibited altered expression of genes associated with phytohormone metabolism specifically in leaves (Figs. 3 and 4, Table S2). Consistent with these transcriptomic changes, phytohormone profiling demonstrated significant perturbations in cytokinin (CK) metabolism in leaves (Fig. 4), whereas no comparable changes were detected in roots (Table S4). Total CK levels (tot-CK) were significantly elevated, primarily due to increased accumulation of O- and N-glucoside conjugates (CK-OG and CK-NG). In contrast, levels of cytokinin ribosides (CK-R) were reduced (Fig. 4b).

**Figure 4:**
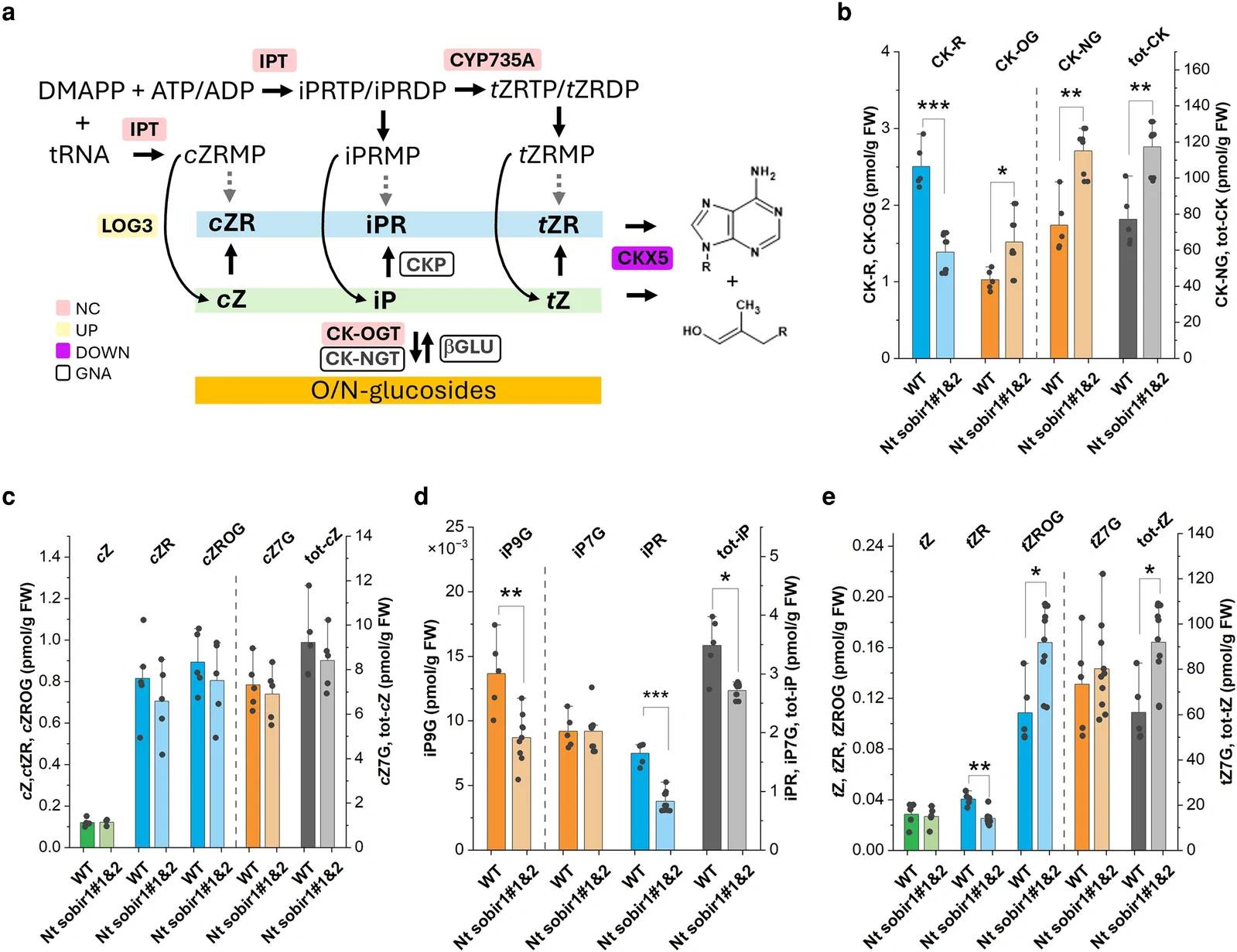
Cytokinin metabolism and profiling in *N. tabacum* WT and *Nt sobir1* double mutant lines. a) Schematic representation of selected cytokinin metabolic pathways showing biosynthesis, interconversion, and degradation steps. Key enzymes are color-coded to indicate transcriptional regulation in *NT sobir1* double mutants (UP-upregulated, DOWN-downregulated, NC-not changed and GNA-gene not annotated). b to e) Quantification of cytokinin metabolites in leaves of WT and 2 independent *NT sobir1* double mutant lines (#1 and #2). b) Glucosylated cytokinin conjugates (CK-R, CK-OG, CK-NG) and total cytokinin pool (tot-CK). c) *cis*-Zeatin-type cytokinins (*c*Z, *c*Z7G, *c*ZR, *c*ZROG) and total *c*Z-type cytokinin content (tot-*c*Z). d) Isopentenyladenine-type cytokinins (iP7G, iP9G, iPR) and total iP-type cytokinin content (tot-iP). e) *trans*-Zeatin-type cytokinins (*t*Z, *t*ZR, *t*ZROG) and total *t*Z-type cytokinin content (tot-*t*Z). Data are presented as means ± Sd (*n* = 5) and were obtained via the LC-MS/MS method. Asterisks indicate statistically significant differences compared with WT (*P* < 0.05 (*), *P* < 0.01 (**), *P* < 0.001 (***); Student's *t*-test). Abbreviations: CYP, cytochrome P450 monooxygenase; CK-NGT, cytokinin *N*-glucosyltransferase; CK-OGT, cytokinin *O*-glucosyltransferase; CKP, cytokinin riboside phosphorylase; CKX, cytokinin dehydrogenase; β-GLU, β-glucosidase; IPT, isopentenyltransferase; LOG, cytokinin phosphoribohydrolase LONELY GUY; *c*Z, *cis*-zeatin; *c*ZR, *cis*-zeatin riboside; *c*ZROG, *cis*-zeatin riboside-*O*-glucoside; *c*Z7G, *cis*-zeatin-7-glucoside; iP, N^6^-isopentenyladenine; iPR, N^6^-isopentenyladenosine; iP7G, N^6^-isopentenyladenine-7-glucoside; iP9G, N^6^-isopentenyladenine-9-glucoside; *t*Z, *trans*-zeatin; *t*ZR, *trans*-zeatin riboside; *t*ZROG, *trans*-zeatin riboside-*O*-glucoside.

Among individual CK types, *trans*-zeatin(*t*Z)-type CKs (tot-*t*Z) represented the predominant form, followed by *cis*-zeatin(*c*Z)-type (tot-*c*Z) and isopentenyladenine(iP)-type (tot-iP) (Fig. 4c to e). The levels of total *t*Z-type CKs and their riboside-O-glucosides (*t*ZROG) were increased, whereas the levels of total iP-type CKs, their N9-glucosides (iP9G), and the riboside forms of *t*Z (*t*ZR) and iP (iPR) were reduced relative to WT plants. In contrast, the concentrations of the biologically active CK bases, *t*Z and *c*Z, remained similar in WT and mutant lines. The changes in endogenous cytokinin levels may be linked to the upregulation of *LOG* family genes (converting CK ribotides to their active CK bases) (Kurakawa et al. 2007) and the downregulation of *CKX*, which irreversibly cleaves unsaturated isoprenoid N^6^ side chains from CK bases and their corresponding ribosides (Bilyeu et al. 2001; Galuszka et al. 2001; Fig. 4a).

In addition, an auxin metabolism was altered, as both mutants displayed increased levels of auxin precursors, tryptamine in leaves and roots, and anthranilic acid in leaves, whereas indole-3-acetic acid (IAA) levels remained unchanged (Fig. S9). Together, these results indicate that NtSOBIR1 loss leads to selective reprogramming of CK and auxin metabolism machinery, mainly in leaves, while maintaining stable levels of bioactive CKs and IAA.

### SOBIR1 maintains immune homeostasis in roots by restraining MAPK–SA signaling and redox pathways

In contrast to leaves, roots of *Nt sobir1 #1* and *#2* double-mutant plants displayed a markedly different transcriptional and phytohormonal profile characterized by constitutive activation of immune-associated pathways. Upregulated genes were significantly enriched in biological processes related to defense response, wounding, and phosphorelay signaling (Fig. 2f, Table S3), accompanied by elevated expression of MAPKs genes *YODA* and *MPK3*, increased accumulation of SA (Fig. 3b), and induction of several SA-responsive genes (acidic endochitinase or glucan endo-1,3-beta-D-glucosidase). Several ethylene-responsive TFs were also induced, consistent with activation of an SA–ethylene-associated immune program. In addition, genes associated with the hydrogen peroxide catabolic process were significantly enriched among downregulated transcripts (Fig. 2g, Table S3), including several class III peroxidases (Peroxidase 3, 10, and 27), indicating altered regulation of ROS homeostasis in roots of *Nt sobir1* double mutant plants. At the same time, although expression of *LOX3* was increased, levels of the jasmonate precursor OPDA were reduced (Fig. 3a), indicating suppression of downstream jasmonate accumulation. Phytohormone profiling confirmed this reciprocal pattern, with SA increased and OPDA decreased in roots (Fig. 3a). Together, these results indicate that NtSOBIR1 normally restrains basal MAPK–SA signaling and redox pathways in roots, and that its loss leads to constitutive activation of SA/ethylene-associated immunity coupled with altered ROS homeostasis.

### The role of SOBIR1 in cell death triggered by elicitins

Together, the above results demonstrate that CRISPR–Cas9-mediated inactivation of SOBIR1 leads to profound tissue-specific rewiring of immune signaling, phytohormone homeostasis, and redox regulation under normal growth conditions. These basal alterations suggest that SOBIR1 functions as a key signaling hub maintaining immune and physiological homeostasis in tobacco. Given the established role of SOBIR1 as a coreceptor for RLPs involved in pathogen perception, we next investigated how loss of SOBIR1 affects inducible immune responses triggered by biotic stress signals, focusing on elicitins, the ExIPs derived from *Phytophthora* species.

In line with findings from *S. microdontum* and *N. benthamiana*, and consistent with the altered basal immune signaling observed in *NT* sobir1 double mutants under nonstress conditions, *Nt sobir1* double mutants failed to induce an HR cell death after infiltration with 5 elicitins from different *Phytophthora (Ph)* and *Pythium* species: infestin (*Ph. infestans*), β-cryptogein (*Ph. cryptogea*), sylvaticin (*Pythium sylvaticum*), capsicein (*Ph. capsici*), and parasiticein (*Ph. parasitica*). However, mutants retained HR induction in response to the elicitor harpin HrpZ_Pph_ from *Pseudomonas syringae* pv. *phaseolicola*, whose receptor remains unidentified (Li et al. 2005; Fig. 5a). As expected, no HR occurred following treatment with the *Xanthomonas* flg22 peptide (Sun et al. 2006; Mani et al. 2023) or the oomycete-derived Pep13 oligopeptide (Halim et al. 2004). To test whether the 2 tobacco SOBIR1 homeologs have an overlapping function, we complemented the *Nt sobir1* double mutant with *NtSOBIR1-1* or *NtSOBIR1-2* individually. Expression of either gene alone restored cryptogein-induced HR/cell death to a comparable extent (Fig. S10), indicating that *NtSOBIR1-1* and *NtSOBIR1-2* are functionally interchangeable in this SOBIR1-dependent immune response. Stable suspension cultures derived from leaves of WT and 2 independent *NT* sobir1 double mutant lines (*Nt sobir1 #1* and *#2*) confirmed these results. Fluorescein diacetate (FDA) viability assays showed strong cell death in WT cultures treated with elicitins, whereas mutants exhibited no significant change (Fig. 5b), stating that SOBIR1 is essential for elicitin-triggered cell death signaling.

**Figure 5:**
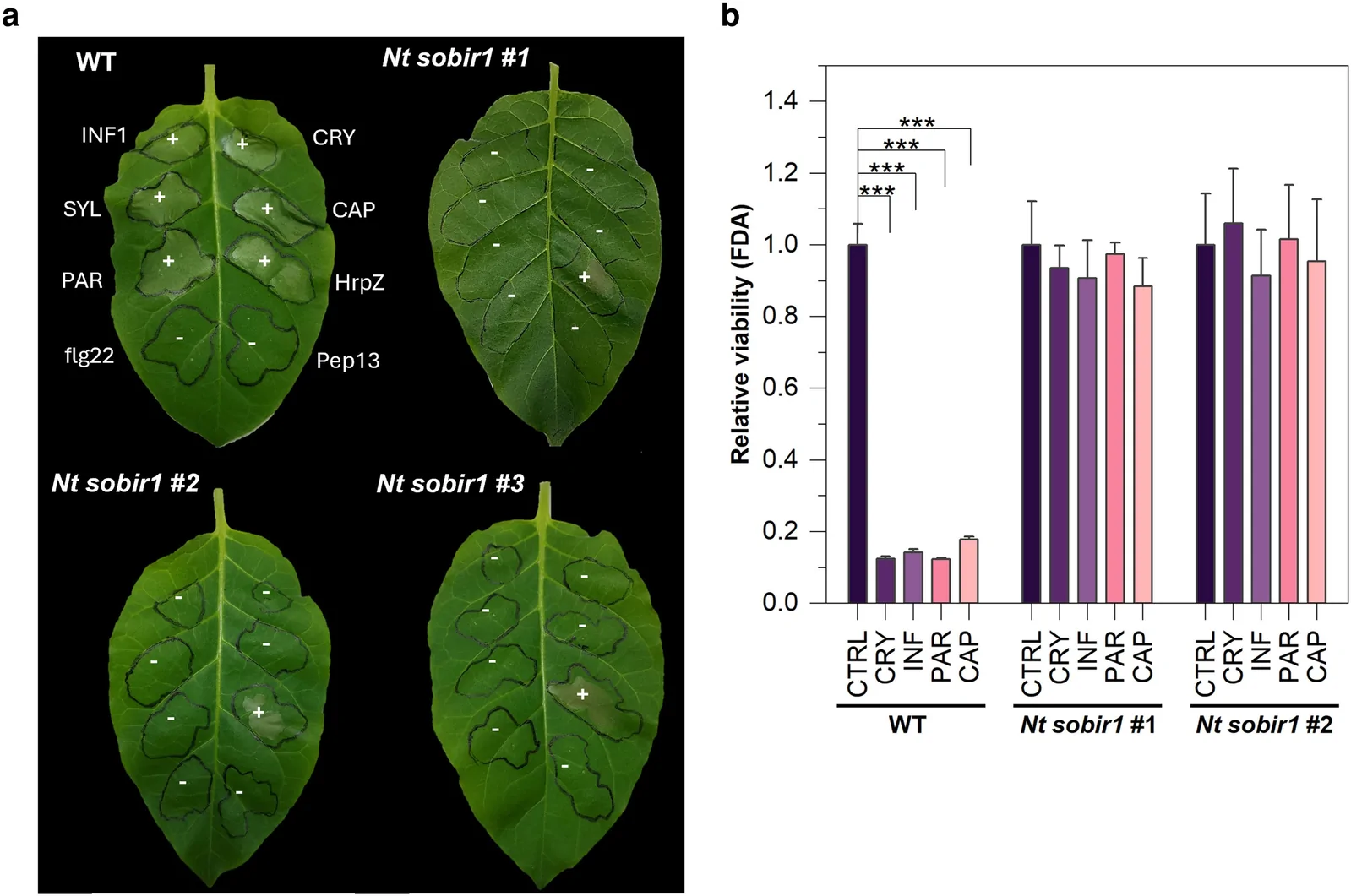
NtSOBIR1 is required for elicitin-induced cell death in tobacco leaves and cell suspensions. a) Cell death responses triggered by various elicitors in WT and *Nt sobir1* double mutant plants. Infiltrated ExIPs included HrpZ protein, peptides flg22 and Pep13, and elicitins produced in *P. pastoris* (CRY, INF1) or purified from the culture media of *Phytophthora* and *Pythium* species (CAP, PAR, SYL). Infiltration zones are marked with black outlines. Cell death symptoms (+) were assessed visually 2 d post-infiltration. The experiment was repeated 3 times with consistent results. b) Quantification of elicitin-induced cell death using the FDA staining method, 24 h after elicitor treatment. Statistical significance was determined via 1-way ANOVA followed by Tukey's post hoc test (*n* = 8). Abbreviations: CAP (capsicein from *Phytophthora capsici*), CRY (β-cryptogein from *Phytophthora cryptogea*), flg22 (flagellin-derived peptide from *Xanthomonas* sp.), HrpZ (harpin from *P. syringae* pv. *phaseolicola*), INF1 (infestin 1 from *P. infestans*), PAR (parasiticein from *P. parasitica*), Pep13 (oligopeptide from an oomycete-derived glycoprotein), SYL (sylvaticin from *P. sylvaticum*).

### Loss of SOBIR1 attenuates the transient ROS response and abolishes sustained ROS accumulation induced by ExIPs

To assess SOBIR1's role in bipartite receptor–mediated ROS production and cell death upon ExIP recognition, we used the elicitin–tobacco cell suspension model (Solanský et al. 2021). In WT cultures, β-cryptogein (β-CRY) induced the characteristic 2-phase ROS burst (Wi et al. 2012) followed by cell death (Fig. 6a). In both *Nt sobir1* double mutant lines, the second, sustained ROS phase and cell death were abolished, while the initial ROS burst remained at ∼50% of WT intensity (Fig. 6a), in agreement with the basal suppression of MAPK components and altered redox-related gene expression observed in *Nt sobir1* plants under normal growth conditions. A similar early ROS response was observed in *Nt sobir1* double mutant suspension cells after elicitation with 2 additional elicitins, infestin and parasiticein, indicating that the residual early response was not specific to β-CRY treatment (Fig. S11a and b). These results show that NtSOBIR1 is not strictly required for initiation of the transient ROS response, although it contributes to its full amplitude. By contrast, sustained ROS accumulation and subsequent HR-like cell death were not detected in the *Nt sobir1* double mutants. Thus, the transient and sustained responses represent functionally distinguishable outputs with different degrees of dependence on NtSOBIR1. Consistent with the preservation of early signaling events in *Nt sobir1* double mutants, transcriptional analysis revealed induction of defense-associated genes, including *WRKY15* and *COMT*, 2 h after β-CRY treatment (Fig. 6b), indicating partial activation of downstream defense responses despite the absence of sustained ROS accumulation and cell death.

**Figure 6:**
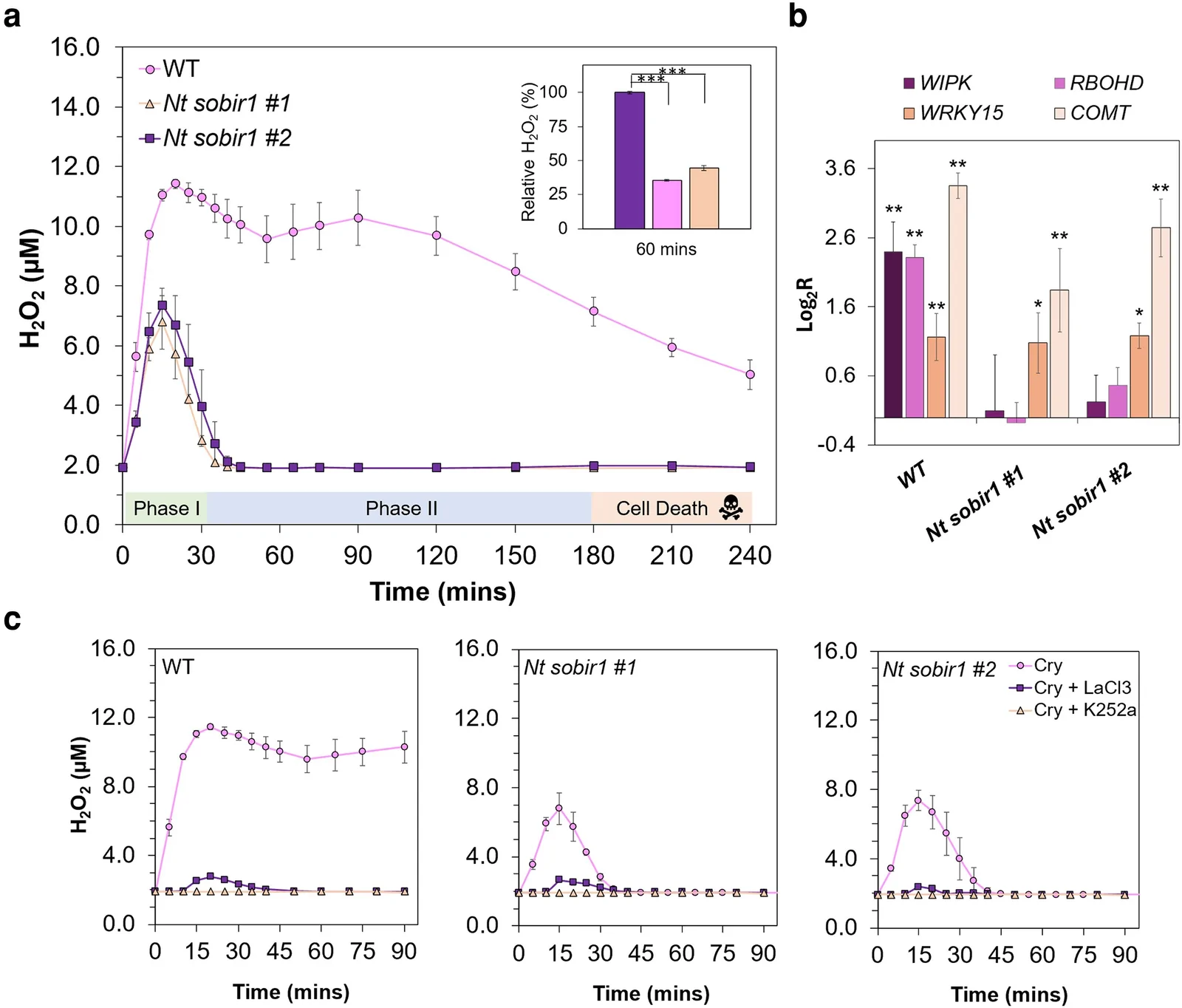
Transient and sustained cryptogein-induced ROS outputs differ in their dependence on NtSOBIR1. a) Time course of ROS (H_2_O_2_) production in 4-d-old cell suspensions elicited with 50 nm CRY, measured using a luminol-based chemiluminescence assay. b) Transcript accumulation of *WIPK*, *RBOHD*, *WRKY15*, and *COMT* at 2 h after β-cryptogein treatment. Gene expression was normalized, and statistical significance was determined using Student's *t*-test (*n* = 3). c) Effects of LaCl_3_ and the nonselective protein kinase inhibitor K-252a on the transient ROS response.

To determine whether this modulatory effect of NtSOBIR1 extends to other ExIPs we examined ROS production following treatment with flg22 (flagellin-derived peptide from *Xanthomonas* sp.) and partially deacetylated chitosan, previously used by Petutschnig et al. (2010), which is directly bound by the LysM receptor kinase CERK1 and induces CERK1-dependent phosphorylation, ROS production, and MAPK activation. Both elicitors triggered ROS production in *Nt sobir1* cell culture (Fig. S11a), indicating that NtSOBIR1 differentially modulates ROS outputs depending on the elicitor context. It is required for full amplification of ExIP-triggered ROS, and it is largely dispensable for canonical flg22 or chitosan-triggered ROS production.

Early ROS formation is known to depend on Ca^2+^ influx, protein phosphorylation (Kadota et al. 2004), and the 48 kDa kinase SIPK (Zhang and Klessig 2001), and is mediated by activation of a preexisting plasma-membrane–resident pool of RBOHD that is rapidly activated upon elicitation, whereas sustained ROS requires redistribution of RBOHD from Golgi-associated intracellular pools to the PM together with de novo RBOHD transcription/translation (Noirot et al. 2014) and activation of the 44 kDa kinase WIPK, correlating with HR cell death (Zhang et al. 2000). Consistently, treatment with the Ca^2+^ channel blocker LaCl_3_ (100 *µ*m) or the nonselective protein kinase inhibitor K-252a (1 *µ*m) (Kadota et al. 2004) suppressed the transient early ROS response in both WT and *Nt* sobir1 double mutants (Fig. 6c), indicating that the residual response in the mutants retains Ca^2+^- and protein kinase-dependent components. These pharmacological data characterize the signaling requirements of the residual ROS output but do not place NtSOBIR1 at a specific position within the pathway. Moreover, β-CRY elicitation induced *WIPK* and *RBOHD* upregulation only in WT cultures (Fig. 6b), consistent with the reduced basal expression of MAPK signaling components observed in *Nt sobir1* double mutants, and supporting an association between SOBIR1-dependent transcriptional activation of WIPK and RBOHD and maintenance of the sustained ROS response.

A transient ROS burst observed in *Nt* sobir1 #1 and *Nt* sobir1 #2 double mutant lines following elicitin treatment may reflect a previously documented attenuated elicitor-induced H_2_O_2_ accumulation in Gα- and Gβ2-silenced *N. benthamiana* plants challenged with the elicitin boehmerin (Zhang et al. 2012). Additionally, direct interaction between NADPH oxidase and the Gβ subunit of the plant heterotrimeric G-protein has been reported, implicating G-protein signaling in NADPH oxidase-mediated ROS production upon elicitin perception (Torres et al. 2013). To assess the role of G-protein-mediated signaling in β-CRY-induced ROS production, tobacco cell suspensions were pretreated with guanosine 5′-[β-thio]diphosphate (GDPβS), a nonhydrolyzable GDP analogue and established inhibitor of G-protein activation (Steffens and Sauter 2009). GDPβS treatment resulted in a ∼40% reduction of ROS accumulation in WT cells and a ∼60% reduction in *Nt sobir1* double mutant lines (Fig. S11b). These data support the involvement of G-protein signaling in the ROS production cascade triggered by elicitin perception in tobacco cells.

### SOBIR1-dependent and independent transcriptional reprogramming of defense responses in tobacco following β-CRY treatment

It was demonstrated that elicitin-induced cell death is strongly impaired in *N. benthamiana sobir1* knockouts, but the extent of residual signaling in *N. tabacum sobir1* mutants was unclear. We observed partial early ROS production in mutant suspension cells after β-CRY treatment, suggesting incomplete defense activation, in line with the preexisting imbalance in immune signaling pathways identified in *Nt sobir1* double mutants under basal conditions. RNA-seq analysis of WT, *Nt* sobir1 *#1*, and *Nt sobir1 #2* plants 18 h after β-CRY treatment identified 3,913 DEGs shared between the 2 *sobir1* mutant lines and 10,835 DEGs in WT plants compared with mock controls (*P* < 0.05; ≥2-fold; Fig. 7a, Table S5). Notably, the majority of β-CRY-responsive genes in *Nt sobir1* double mutants overlapped with those regulated in WT plants, indicating substantial conservation of the transcriptional response. This indicates that β-CRY perception triggers a largely conserved early transcriptional defense program in *Nt sobir1* double mutants, despite the absence of sustained ROS production and HR-like cell death. In mutants, upregulated DEGs were enriched for biological processes related to phenylpropanoid and porphyrin metabolism and light response, suggesting a coordinated shift from growth to enhanced immune readiness (Fig. 7b, Table S6). Upregulated light-response genes included phytochrome B, which promotes SA- and JA-mediated defenses (Kim et al. 2021), and gibberellin (GA) 2-beta-dioxygenase, which inactivates GA (Thomas et al. 1999), stabilizing DELLA proteins and enhancing JA responses. Increased expression of porphyrin metabolism genes involved in chlorophyll biosynthesis (eg chlorophyllide a oxygenase) (Reinbothe et al. 2006) and degradation (eg chlorophyllase-2, stay-green proteins) (Sakuraba et al. 2012) suggests β-CRY triggers chloroplast remodeling to enable controlled ROS production and retrograde signaling while preventing premature senescence. This was accompanied by aquaporin gene induction, supporting ROS transport, SAR, cell wall reinforcement (Tian et al. 2016; Zhang et al. 2019), maintenance of water balance (Kapilan et al. 2018), and activation of phenylpropanoid metabolism (eg *PAL*). In contrast, downregulated DEGs were mainly linked to the biological processes related to RNA metabolism (Fig. S12, Table S6), including many pentatricopeptide repeat proteins (Wang and Tan 2025), and circadian rhythm regulation (eg *gigantea*, *early flowering 4*) (Kim et al. 2013; Mishra and Panigrahi 2015), consistent with suppression of chloroplast and mitochondrial activity to prioritize defense, a hallmark of immune responses (Table S6). Although glucan catabolic process terms were enriched among downregulated DEGs after β-CRY treatment (Fig. S12, Table S6), the affected genes primarily encoded enzymes involved in starch and α-glucan mobilization (eg α-1,4-glucan phosphorylase, isoamylase 3, DSP4, and α-amylase 3), suggesting suppression of carbohydrate turnover as part of cryptogein-induced metabolic reprogramming.

**Figure 7:**
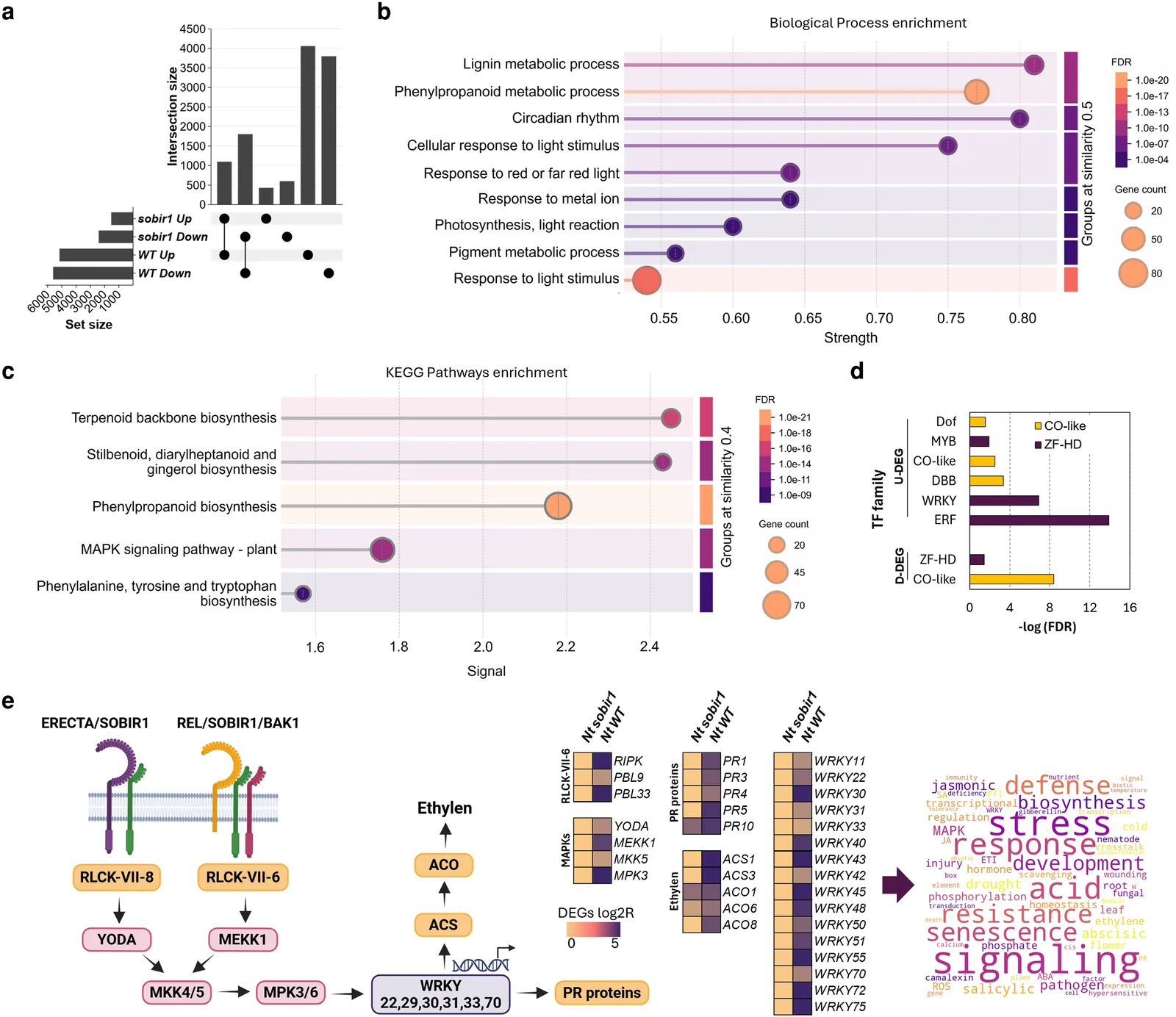
Elicitin-induced transcriptomic changes in leaves of *N. tabacum sobir1*-double mutant lines (*Ntsobir1*) and WT plants. a) UpSet plot summarizing DEGs identified in WT and *Nt sobir1* plants following treatment with the elicitin β-cryptogein (β-CRY). The horizontal bar chart (left) shows the total number of DEGs detected in each genotype, and the vertical bar chart (top) displays the size of DEG intersections across genotypes, as indicated by the intersection matrix below. b) STRING Gene Ontology (GO) Biological Process enrichment dot plot of genes commonly upregulated (fold change > 2, *P*adj < 0.05) in both *Nt sobir1* #1 and #2 double mutants after β-CRY treatment. GO term descriptions are shown on the *y*-axis, enrichment strength on the *x*-axis, dot size indicates the observed gene count, and color represents the FDR, as defined by STRING. c) STRING KEGG pathway enrichment dot plot of genes upregulated (fold change > 2, *P*adj < 0.05) after β-CRY treatment in WT compared with both *Nt sobir1* #1 and #2 double mutants. KEGG pathway descriptions are shown on the *y*-axis, enrichment strength on the *x*-axis, dot size indicates the observed gene count, and color represents the FDR. d) Enrichment of TF families among upregulated (U-DEG) and downregulated (D-DEG) genes in WT and *Nt sobir1* plants, identified by chi-squared analysis. e) Schematic representation of genes significantly upregulated (fold change > 2, *P*adj < 0.05) after β-CRY treatment and involved in the MAPK signaling pathway, activated downstream of the LRR-RLK SOBIR1 complex. Also shown is a word cloud generated from functional terms and biological processes associated with WRKY TFs upregulated exclusively in WT plants, based on literature-based text mining.

To directly compare β-CRY-induced transcriptional responses between WT and *Nt sobir1* double mutant plants, we analyzed DEGs following β-CRY treatment. In β-CRY-treated WT tobacco leaves, a distinct set of upregulated DEGs demonstrated the essential role of SOBIR1 in activating MAPK signaling cascades, which are already compromised in *Nt sobir1* double mutants under noninduced conditions. In WT plants, MAPK activation led to strong upregulation of WRKY TFs (Fig. 7c and d, Table S6), which regulate ethylene biosynthesis genes (*ACS*, *ACO*) and pathogenesis-related (PR) genes (Fig. 7e; Javed and Gao 2023), supporting biological processes such as stress response, immune activation, and pathogen resistance. Further analysis revealed robust induction of ethylene-responsive TFs (ERFs, Fig. 7d), particularly Group IX AP2/ERFs, known to be differentially induced by ET, JA, and SA (Feng et al. 2020). This group included positive regulators (ERF1, ERF2, ERF13, ERF14, ERF096, ERF098, ERF109, ERF113), which bind GCC-box motifs in defense gene promoters to activate JA/ET-dependent transcriptional reprogramming and contribute to HR-like responses. Repressors (ERF3, ERF4) containing EAR motifs were also upregulated, suggesting that SOBIR1 signaling both activates and fine-tunes immune responses (Ohta et al. 2000; Wu et al. 2022). Many of these ERFs are induced independently of ethylene, relying instead on early MAPK activation, indicating a phytohormone-independent entry point into the defense network. HR-like cell death in WT plants coincided with strong upregu^l^ation of *LOX5* and *PAT2/PAT3*, associated with JA-independent oxylipin signaling and HR induction (Dhondt et al. 2002). The SOBIR1-mediated cascade was also linked to activation of KEGG pathways for plant–pathogen interaction, aromatic amino acid biosynthesis, glycolysis and the TCA cycle, and terpenoid biosynthesis (Table S6). These shifts suggest increased energy and precursor demand to support synthesis of defense metabolites, phytohormones, and stress compounds such as glutathione, arginine, and proline.

### The role of SOBIR1 in pathogen resistance in tobacco

To assess SOBIR1's role in pathogen defense, 2 *sobir1* double mutant lines were tested against 3 pathogens with distinct lifestyles: *P. syringae* pv. *tabaci* (biotroph), *P. parasitica* (hemibiotroph), and *Botrytis cinerea* (necrotroph). As in SOBIR1-knockout *N. benthamiana* (Huang et al. 2024), both mutants showed increased susceptibility to *P. parasitica* (Fig. 8a), but no difference in susceptibility to *B. cinerea* or *P. syringae* (Fig. 8b and c). To test SOBIR1's involvement in elicitin-mediated systemic resistance, WT and *Nt sobir1* double mutant plants were pretreated with β-CRY 24 h before inoculation with all 3 pathogens. Only in the case of *P. parasitica* and *P. syringae* β-CRY enhanced resistance in WT but not in mutants (Fig. 8), indicating NtSOBIR1 is essential for immune responses to specific ExIPs such as elicitins, but not for general pathogen resistance in *N. tabacum*, thereby linking its inducible immune function to its role in maintaining basal immune homeostasis.

**Figure 8:**
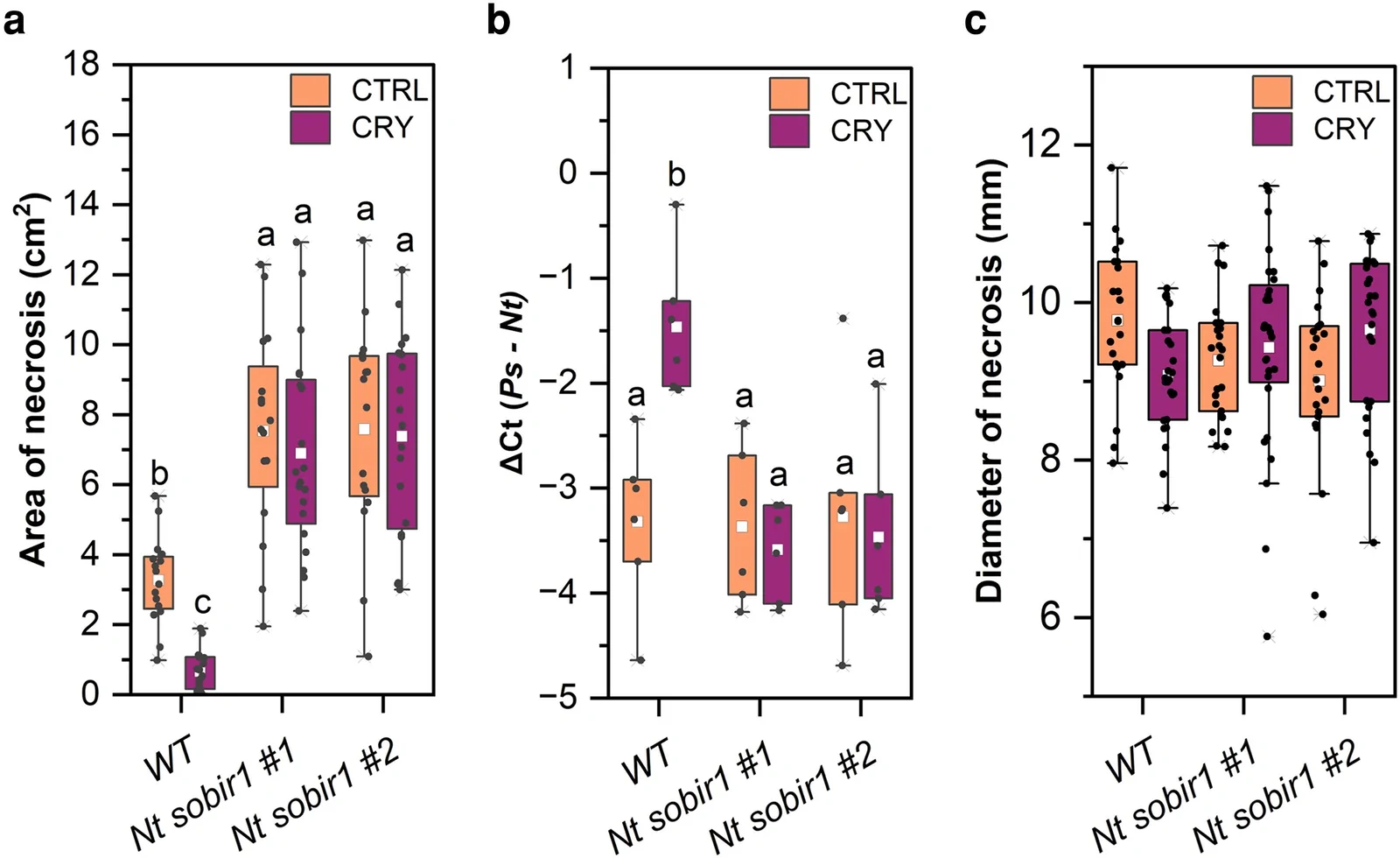
Resistance levels of *Nt sobir1* double mutant lines against diverse pathogens. All analyses were carried out on 7-wk-old *N. tabacum* plants. Resistance to individual pathogens was assessed in leaves from control plants (CTRL) and decapitated plants treated with the elicitin β-cryptogein (CRY, 5 *µ*g) 24 h before pathogen inoculation. a) Resistance to *P. parasitica* was assessed by inoculating leaves with zoospores. The invaded areas were measured 72 h post-inoculation. Each bar represents the standard error of 4 replicates from 3 independent experiments. Each replicate consisted of 8 inoculated sites on 4 leaves from a single plant. b) Resistance to *P. syringae* pv. *tabaci* was assessed by inoculation with *P. syringae* pv. *tabaci* via direct syringe infiltration into the leaf apoplast. Resistance levels were quantified after 72 h using qPCR and evaluated via the Δ*Ct* method, with the EF1α gene from tobacco (*Nt*) and the PSPTO_4345 gene (GenBank: CP047072) from *P. syringae* pv. *tabaci* (*Ps*). c) Resistance to *B. cinerea* was on leaf discs inoculated with *B. cinerea* strain BBM1 (6 *µ*L of a 5 × 10^4^ conidia/mL suspension), and lesion areas were measured 48 h post-inoculation. Values represent the mean ± Sd of at least 50 lesions.

In all measurements, statistical significance was determined using 1-way ANOVA followed by Duncan's post hoc test. Different lowercase letters indicate significant differences (*P* ≤ 0.05).

## Discussion

SOBIR1 is widely recognized as a signaling RLK that constitutively associates with LRR-RLPs to form bipartite receptor complexes, in which the RLP provides extracellular ligand recognition, and SOBIR1 provides intracellular kinase activity (Liebrand et al. 2014; Domazakis et al. 2018). Our results support this model in tobacco but refine it by showing that the contribution of SOBIR1 to immunity is highly context dependent. NtSOBIR1 was indispensable for the full immune response triggered by oomycete elicitins, yet it was not required for all early defense outputs or for resistance against all tested pathogens. Thus, rather than acting as a general determinant of pathogen resistance, NtSOBIR1 appears to be specifically required in host–pathogen interactions where effective immunity depends on recognition of particular ExIPs by SOBIR1-associated receptor complexes.

Because allotetraploid tobacco contains 2 expressed *NtSOBIR1* homeologs, both copies had to be inactivated to assess SOBIR1 function reliably. Complementation with either *NtSOBIR1-1* or *NtSOBIR1-2* restored the β-cryptogein-induced cell death, showing that the 2 homeologs are functionally interchangeable in this response and validating the double mutant as a robust system for studying SOBIR1-dependent signaling. Using this system, we found that loss of both *NtSOBIR1* genes abolished cell death induced by several elicitins, including β-cryptogein whereas the response to the unrelated bacterial elicitor HrpZ was retained. These results are consistent with previous work showing that elicitins are recognized by REL/ELR-type LRR-RLP receptors that associate with SOBIR1 and BAK1/SERK-family co-receptors in Solanaceae (Liebrand et al. 2013; Du et al. 2015; Domazakis et al. 2018; Chen et al. 2023), and they place NtSOBIR1 as an essential signaling component of the elicitin receptor pathway in tobacco.

A key finding of this study is that SOBIR1 is not required for all elicitin-induced signaling events. In *Nt sobir1* cell suspensions, β-cryptogein still triggered an early ROS burst, although at reduced amplitude, whereas the sustained ROS phase and subsequent cell death were abolished. This kinetics pattern resembled that observed in *Cf-4/rlck-vii-6* mutants of *N. benthamiana*, which show an attenuated early phase and a complete loss of the prolonged ROS response (Huang et al. 2024). In *N. tabacum*, RBOHD is the principal source of apoplastic ROS during plant–microbe interactions (Simon-Plas et al. 2002), while sustained ROS accumulation depends on transcriptional upregulation of *RBOH* genes through WRKY TFs, including *Nt*WRKY70 (Adachi et al. 2015; Zhang et al. 2024). Consistent with this model, β-CRY failed to induce *NtWRKY*70 in *Nt sobir1* double mutants. Similarly, transcripts encoding RLCK-VII kinases, including members of the VII-6 subclass (eg RIPK and PBL33) and the VII-8 subclass (eg PBL9), were not induced. These subclass assignments are based on phylogenetic comparison with previously characterized *N. benthamiana* RLCK-VII members (Fig. S13). These kinases are known to regulate RBOH activation through phosphorylation and contribute to resistance against *Phytophthora* spp. (Kadota et al. 2019; Liang et al. 2021; Huang et al. 2024), although their relative roles may differ between species, possibly reflecting differences in RBOH isoform usage (Simon-Plas et al. 2002; Yoshioka et al. 2003; Huang et al. 2024).

Together, these observations identify 2 functionally distinguishable kinetic outputs following elicitin perception in tobacco. In WT cells, a transient ROS peak was followed by sustained ROS accumulation and HR-like cell death. In the *Nt sobir1* double mutants, the transient response was attenuated but retained, whereas sustained ROS accumulation, induction of *WIPK* and *RBOHD*, and HR-like cell death were absent. The 2 outputs therefore differ in their dependence on SOBIR1. However, the present experiments do not establish whether they arise from separate signaling branches or represent temporally distinct and differentially amplified states of an interconnected signaling network.

As a working model, we propose that SOBIR1 promotes the amplification and/or maintenance of cryptogein-induced signaling required for sustained ROS accumulation and HR-like cell death, whereas initiation of the transient ROS response can occur through signaling components that remain functional in its absence. Previous work demonstrated that the elicitin receptor REL associates with SOBIR1 and BAK1 in planta even in the absence of ligand, consistent with the potential existence of preassembled receptor complexes (Chen et al. 2023). By analogy with the Cf-4/SOBIR1/BAK1 complex, β-CRY perception may promote SOBIR1 autophosphorylation and reciprocal *trans*-phosphorylation between SOBIR1 and BAK1, potentially coupling receptor activation to downstream RLCK-mediated signaling (Huang et al. 2024). In the absence of SOBIR1, limited signaling through BAK1-associated or partially redundant components could contribute to the residual transient ROS response. BAK1 activity is subject to negative regulation by protein phosphatase 2A (Segonzac et al. 2014), although whether changes in this regulation contribute to the response retained in the mutants remains unknown. Furthermore, the inhibition of β-CRY-induced ROS accumulation by GDPβS, together with the previously reported involvement of Gα and Gβ2 in elicitor-induced H_2_O_2_ accumulation in *N. benthamiana* (Zhang et al. 2012), is consistent with a contribution of heterotrimeric G-protein-related signaling. However, these observations do not establish its specific role in the residual response or its mechanistic relationship with SOBIR1. Thus, this model accommodates functionally distinguishable transient and sustained ROS outputs without requiring separate signaling branches; the molecular components supporting residual signaling and the mechanisms underlying SOBIR1-dependent amplification remain to be established through direct genetic and biochemical analyses.

The pathogen assays further demonstrate that SOBIR1-dependent immunity in tobacco is pathogen-specific. *Nt sobir1* mutants were more susceptible to *P. parasitica*, and β-cryptogein pretreatment enhanced resistance in WT plants but not in the mutants. In contrast, basal resistance to *B. cinerea* and *P. syringae* pv. tabaci was not significantly altered. These findings indicate that SOBIR1 is crucial when resistance depends on a SOBIR1-associated elicitor recognition system, as in the elicitin–*Phytophthora* interaction but is not universally required for effective defense. The absence of a strong phenotype against *B. cinerea* is consistent with observations in *N. benthamiana*, where *sobir1*- or *bak1*-silenced plants did not show enhanced susceptibility to this pathogen, even when challenged with strains deficient in major phytotoxic compounds (Leisen et al. 2022). Although SOBIR1 has been linked to fungal elicitor perception and RLP30-mediated responses in Arabidopsis (Zhang et al. 2013, 2014), these responses do not always translate into increased quantitative resistance. Thus, SOBIR1-dependent signaling is critical for resistance to specific apoplastic ExIPs-secreting biotrophic and hemibiotrophic pathogens, such as *C. fulvum* and Phytophthora spp. (Liebrand et al. 2013; Du et al. 2015; Chen et al. 2023), whereas its contribution to defense against necrotrophic fungi appears to be species- and context-dependent. This context dependence is also evident from the responses to other elicitors. ROS production induced by flg22 or chitosan was retained in *Nt sobir1* suspension cultures, indicating that SOBIR1 is largely dispensable for canonical FLS2- or CERK1-associated early ROS responses (Chinchilla et al. 2006; Petutschnig et al. 2010). Therefore, SOBIR1 does not disturb RBOH activity. Instead, it is specifically required for full amplification of ROS outputs downstream of particular ExIP receptor systems, especially those involving LRR-RLP/SOBIR1 complexes. This narrower interpretation reconciles the strong elicitin phenotype with the more restricted pathogen-resistance phenotype and positions SOBIR1 as a receptor-complex component that determines whether specific extracellular signals are translated into sustained immune activation.

Although the strongest phenotype of *Nt sobir1* mutants concerns elicitin-triggered immunity, the basal transcriptomic and physiological changes suggest that SOBIR1 also contributes to immune homeostasis under noninduced conditions. In leaves, SOBIR1 loss was associated with reduced expression of several immune-related MAPK components and lower SA levels, whereas JA and OPDA accumulated. In roots, by contrast, SOBIR1 loss was associated with activation of MAPK–SA-related defense signatures and altered redox homeostasis. These tissue-specific effects indicate that SOBIR1 participates in maintaining basal immune balance, but they are unlikely to be the primary explanation for the pathogen-resistance phenotypes. If phytohormone changes alone determined general resistance, broader susceptibility would be expected. Instead, altered phytohormone metabolism likely modifies the physiological background on which specific SOBIR1-dependent receptor pathways operate. The developmental and phytohormonal changes observed in *Nt sobir1* mutants are consistent with known roles of SOBIR1-containing complexes beyond immunity, including receptor systems involved in organ development, meristem regulation, and cell differentiation (Leslie et al. 2010; Liebrand et al. 2013; Pan et al. 2016). In particular, SOBIR1 has been linked to CLV2-associated signaling, which contributes to meristem maintenance and defense-related responses (Liebrand et al. 2013; Pan et al. 2016), and to ERECTA-related signaling, including the ERECTA–SOBIR1 module involved in xylem fiber differentiation (Milhinhos et al. 2019). CLAVATA signaling is also connected to cytokinin homeostasis in meristems (Meng et al. 2012; Cammarata et al. 2022), whereas the ERECTA–YODA–MKK4/5–MPK3/6 cascade regulates cell proliferation, cytokinin responsiveness, and auxin transport (Meng et al. 2012; Smékalová et al. 2014). Nevertheless, these effects should be interpreted cautiously in our system. *Nt sobir1* mutants did not show major rosette growth defects under the tested conditions, and changes in cytokinin conjugates and auxin precursors occurred without corresponding changes in active cytokinin bases or IAA levels. Thus, the observed phytohormonal and transcriptional changes may reflect altered receptor signaling balance, tissue-specific stress status, or compensatory responses, rather than a separate primary function equivalent to the SOBIR1-dependent elicitin receptor pathway.

The cell wall phenotype provides the clearest example of such a compensatory basal change. *Nt sobir1* double mutants showed increased cell wall stiffness despite induction of cell wall-loosening genes, including expansins, endoglucanases, and pectate lyases, suggesting compensatory cell wall remodeling rather than simple wall loosening. This interpretation is consistent with previous links between SOBIR1 and cell wall-related developmental processes, including the ERECTA–SOBIR1 module acting downstream of BREVIPEDICELLUS in xylem fiber differentiation (Milhinhos et al. 2019), as well as with a role for SOBIR1 in cell wall integrity signaling (Baez et al. 2022). Although expansins are classically associated with relaxation of the cellulose–xyloglucan matrix (Cosgrove 2016), their mechanical effects depend on wall context; for example, EXPA1 overexpression in Arabidopsis can increase cell wall stiffness, potentially through altered pectin remodeling and methylesterification (Levesque-Tremblay et al. 2015; Sénéchal et al. 2015; Samalova et al. 2023). Similarly, expansin induction in *Nt sobir1* double mutants may reflect altered cell wall sensing that reinforces, rather than loosens, the wall matrix. Such remodeling could affect basal defensive preparedness or pathogen penetration, but it did not compensate for the loss of elicitin-triggered resistance to *P. parasitica*. Thus, cell wall remodeling should be viewed as a parallel consequence of SOBIR1 loss that may shape basal resistance, but not as the central mechanism underlying the elicitin phenotype.

Taken together, our results support a model in which NtSOBIR1 has 2 related but distinct roles. First, as part of a bipartite LRR-RLP/RLK receptor module, it is essential for full elicitin-triggered immunity, including sustained ROS production, HR-like cell death, transcriptional amplification, and resistance to *P. parasitica*. Second, under basal conditions, SOBIR1 contributes to tissue-specific immune response, phytohormonal content, and cell wall organization. These basal changes may influence the defensive state of the plant, but they do not make SOBIR1 a universal resistance factor. Instead, the requirement for SOBIR1 becomes evident only in specific host–pathogen interactions where pathogen recognition depends on SOBIR1-associated receptor complexes. This model can explain why *Nt sobir1* mutants show a strong defect in elicitin-triggered responses and Phytophthora resistance, while retaining early ROS signaling and effective resistance to other pathogens.

## Materials and methods

### NtSOBIR1 knockout by CRISPR–Cas9 editing

To generate *Nt sobir1* double mutants, 6-wk-old greenhouse-cultivated bleach-sterilized leaf discs of *N. tabacum* cv. Xanthi were transformed using *Agrobacterium tumefaciens* strain C58 carrying the pDGE792/*SOBIR1-1*-sgRNA/*SOBIR1-2*-sgRNA vector (Stuttmann et al. 2021). This vector was assembled using the Golden Gate Assembly tool and contained 2 distinct gRNAs targeting each SOBIR1 homeolog. gRNA spacer sequences targeting SOBIR1 genes were designed with CRISPOR software (http://crispor.tefor.net/, Fig. S14). The transformation was performed through the co-cultivation method, and Basta-resistant F_0_ transformants were genotyped using PCR, RFLP, and sequencing approaches. F_1_ lines were germinated on sand supplemented with ½ MS medium containing 2 mm 5-fluorocytosine, which served as a negative selection marker for the presence of the Cas9 cassette (Fig. S14). The selected F_1_ individuals underwent further genotyping through PCR and Sanger sequencing, and homozygous double mutants carrying frameshift mutations in both *NtSOBIR1* genes were cultivated to obtain F_2_ seeds. Due to the high nucleotide sequence similarity between *NtSOBIR1-1* and *NtSOBIR1-2*, repeated CRISPR–Cas9 attempts to generate locus-specific single mutants were unsuccessful, as guide RNAs designed against one locus frequently targeted both homologs. Consequently, functional analyses were performed using independent double-knockout lines.

### Plant and cell suspension cultivation

*N. tabacum* cv. Xanthi plants were cultivated in phytotron climate chambers at 24 °C with a 16-h light period with an intensity of 140 *μ*mol m^−2^ s^−1^, and all experiments were conducted using 7-wk-old plants. In vitro cultivated mutant tobacco lines (obtained from sterilized seeds) were employed to induce callus formation by transferring leaf discs to plates containing Murashige and Skoog (MS) cultivation medium [4.41 g/L MS salt medium including Nitsch vitamins (Duchefa, Netherlands), 200 mg/L glutamine, 30 g/L sucrose, 0.1 mg/L kinetin, 0.16 mg/L 2,4-D, 3 mg/L Calcium Pantothenate, Plant agar 8 g/L, pH = 5.7]. After 6 wk, calluses were transferred to the same medium (without agar), and suspensions were subcultured weekly.

### Elicitins preparation

The β-elicitin cryptogein (β-CRY) and α-elicitin infestin 1 (INF1) were expressed in *Pichia pastoris* GS115 and purified as described previously (Sandor et al. 2016). The elicitins capsicein (CAP), parasiticein (PAR) and sylvaticin (SYL) were purified according to Bonnet et al. (1996) from the culture media. All elicitins were dissolved in water as 100 *µ*mol/L stock solutions.

### Phylogenetic analysis

TBlastN analyses were run against NCBI solanaceae genomes (Fig. S1) using *A. thaliana* SOBIR1 protein (GenBank accession number NP_180747.1) as a query sequence. Genomic contexts of blast hits with a minimum of 50% query coverage and 50% sequence identity were manually inspected using the NCBI genome browser to rule out pseudogenes. As a result, 160 SOBIR1 homologs (Fig. S1) were retrieved from the NCBI database and submitted to phylogenetic analysis on the NGPhylogeny.fr web server (https://ngphylogeny.fr/) (Lemoine et al. 2019). A multiple sequence alignment was created using MAFFT, and the informative regions were selected by BMGE using default settings. FastME was employed for tree inference with bootstrap replicates set at 1,000. iTOL v7 was used to visualize and decorate the tree (https://itol.embl.de/) (Letunic and Bork 2024).

### RNA-seq and differential expression analysis

Total RNA was extracted using TRIzol reagent (Life Technologies, Grand Island, NY, USA), DNA-depleted, and quality-checked (RIN ≥ 7.0). Poly(A)+ RNA libraries were prepared using NEBNext kits (NEB, UK) and sequenced on the MGI DNBSEQ-G400 platform (PE150). Reads were trimmed, rRNA-filtered, and aligned to the *N. tabacum* TN90 genome (GCF_000715135.1) (Sierro et al. 2014). Differential expression was assessed using DESeq2 (Love et al. 2014), with DEGs defined as |log_2_FC| > 1 and *q* < 0.01. Enrichment analyses of GO and KEGG terms were performed using ClueGO (v.2.5.10) (Bindea et al. 2009) in Cytoscape (v.3.10.2) (Shannon et al. 2003). A word cloud analysis of WRKY-related biological functions was generated via text mining of scientific literature using the WordCloud Python package. Raw data are available at GEO (GSE307383).

### Elicitin-induced cell death

Individual ExIPs were syringe-infiltrated into leaves from the abaxial side using the following final concentrations: 50 nm β-cryptogein, 75 nm INF1, 300 nm sylvaticin, 50 nm capsicein, 100 nm parasiticein, 5 *µ*m Harpin HrpZPph, 1 *µ*m flg22 peptide, and 10 *µ*m Pep-13. Treated leaves were photographed 24 h post-infiltration to document macroscopic symptoms.

For suspension cell assays, cell death was induced by adding elicitins to the cultivation medium at the following concentrations: 10 nm β-cryptogein, 15 nm INF1, 60 nm sylvaticin, 10 nm capsicein, and 20 nm parasiticein. After 24 h, cell viability was assessed using the FDA staining method. Briefly, 100 *µ*L of treated cell suspension was transferred to a well of a 96-well microplate, followed by the addition of 10 *µ*L of FDA solution (50 *µ*g/mL). Fluorescence was measured 10 min after staining using excitation at 494 nm and emission at 520 nm.

### ROS assay in cell suspensions

ROS production in response to 10 nm β-cryptogein, 1 *µ*m flg22 peptide from *Xanthomonas* sp. and 25 *µ*g L^−1^ chitosan from shrimp shells (Merck C3646, ≥75% deacetylated) was measured using a luminol-based chemiluminescence assay according to (Leborgne-Castel et al. 2008). Where indicated, chemical inhibitors (100 *µ*m LaCl_3_, 1 *µ*m K-252a, or 500 *µ*m GDP-βS) were added 10 min before β-cryptogein treatment to assess their effect on ROS generation. Luminescence was recorded over 90 min using a Berthold Sirius Luminometer (Berthold Technologies, Germany). Values obtained after a 4 s integration of relative luminescence units were expressed as H_2_O_2_ equivalents in *µ*m, using an H_2_O_2_ calibration curve.

### Pathogen infection assays in tobacco

*B. cinerea* (BBM1 strain) was cultured on V8/2 agar medium under dark conditions for 2 wk to induce sporulation (Zimmerli et al. 2001). Conidia were harvested in sterile water, counted using a hemocytometer, and adjusted to a final concentration of 5 × 10^4^ conidia/mL in 0.6% potato dextrose broth. For infection assays, 6 *µ*L of conidial suspension was applied to 2 cm-diameter leaf discs from *N. tabacum* cv. Xanthi plants. Discs were placed on moist filter paper and incubated in darkness at room temperature for 72 h. Disease severity was quantified by measuring lesion diameters using a digital caliper.

For *P. parasitica* infections, zoospore suspensions were prepared according to established protocols (Uhlíková et al. 2016). Approximately 100 motile zoospores (in 50 *µ*L sterile water) were infiltrated into the parenchyma of fully expanded leaves from 7-wk-old plants using a pipette. Two inoculation sites were used per leaf on at least 4 leaves per plant, with 3 independent biological replicates per treatment. Symptom development was assessed 72 h post-inoculation by measuring the lesion area as an indicator of pathogen growth (Galiana et al. 1997; Hugot et al. 1999).

To assess resistance to *P. syringae* pv. *tabaci* (DSM 50311), bacteria were grown in GYE medium at 28 °C and adjusted to 1 × 10^6^ CFU/mL in 10 mm MgCl_2_. The suspension was infiltrated into the abaxial side of tobacco leaves using a needleless syringe. Bacterial proliferation was quantified 48 h post-inoculation by qPCR using gene-specific primers for both the pathogen (*PSPTO_4345*) and host (*EF1α*). Genomic DNA was extracted from infected tissue using the CTAB method (Porebski et al. 1997). Relative bacterial load was calculated using the Δ*Ct* method (Livak and Schmittgen 2001).

For SAR experiments, 20 *µ*L of 250 ng/*µ*L β-cryptogein was applied to the decapitated stem 24 h before pathogen challenge. Subsequent pathogen growth was analyzed as described above.

### Quantification of phytohormones

The quantitative analysis of plant hormones was carried out using an Acquity I-Class UPLC ultra-high performance liquid chromatography system (Waters, Milford, CT, USA) and Xevo TQ-XS tandem mass spectrometer (Waters, Manchester, UK). The sample preparation, chromatography conditions, and mass spectrometer were set according to a previously published protocol (Šimura et al. 2018). Briefly, the tobacco leaves and roots (15 mg FW) were extracted in 1 mL of 60% (v/v) acetonitrile in the presence of stable isotopically labeled internal standards of targeted plant hormones. Four zirconium oxide homogenization beads were added, and the samples were shaken in a Retsch MM400 oscillation mill for 5 min (Retsch, Haan, Germany). Sonication for 5 min and incubation for 30 min at 4 °C in a refrigerator supported the extraction efficiency. Afterwards, the samples were centrifuged at 20,000 rpm, 4 °C for 15 min using an Allegra 64R benchtop centrifuge (Beckman Coulter, USA). The supernatant was collected and purified with Oasis HLB extraction cartridges 30 mg/1 cc (Waters). The flow-through fraction was collected and evaporated to dryness using a SpeedVac concentrator (RC1010 Centrivap Jouan, ThermoFisher, USA). The dry sample was reconstituted in 40 *µ*L of 25% (v/v) acetonitrile and transferred into an LC vial (Chromservis Ltd., Czech Republic). A volume of 5 *µ*L was injected into an Acquity UPLC CSH TM C18 2.1 × 150 mm, 1.7 µm chromatographic column. Data was acquired in multiple reaction monitoring mode. The concentration of plant hormones was calculated using the isotope dilution method (Rittenberg and Foster 1940).

### Analysis of ethylene production

Two fully expanded leaves of *N. tabacum* WT, *Nt sobir1 #1*, and *Nt sobir1 #2* double mutant plants were collected for analysis. From each plant, 2 leaves of similar size were excised, weighed, and placed into 20 mL transparent chromatography crimp vials (Agilent Technologies, USA) containing 2 mL of distilled water. The vials were then sealed and incubated for 24 h in a controlled phytochamber (polyklima M4Z-TDL+, Polyklima, Germany) under the following conditions: 16-h light/8-h dark cycle, 23 °C day temperature, 18 °C night temperature, white light intensity of 110 to 115 *µ*mol m^−2^ s^−1^, and 50% relative humidity. Following incubation, ethylene measurements were performed using a photoacoustic ethylene detector (ETD-300, Sensor Sense, Netherlands) equipped with a VC-6 valve controller (Sensor Sense). Ambient air was used as the carrier gas, supplied via an air pump, filtered to 0.01 *µ*m, and purified from hydrocarbons using a catalytic converter (Sensor Sense). An in-house sampling needle system was employed for gas extraction. Measurements were conducted in sample mode, with the sensitivity set to “fine.” Airflow was maintained at 5 L h^−1^, and each sample was measured over a 5-min sampling period.

### AFM measurements

AFM measurements were conducted using a bio-AFM NanoWizard 3 AFM (Bruker-JPK, Berlin, Germany) combined with an inverted light microscope IX-81 (Olympus, Germany). Before each experiment, the laser reflection sum was maximized, and the laser detector was centered. Immediately after, probe sensitivity and stiffness were determined using the thermal tune method in Bruker-JPK software. The experiment was conducted on hypocotyls of 7-d-old seedlings in a 10% mannitol solution, using an MLCT-BIO-DC-F (Bruker, CA, USA) cantilever. AFM settings were kept constant for each sample, with a setpoint of 3 nN relative to the baseline, Z-length at 3 *µ*m, recording speed at 10 *µ*m/s, and sample rate at 10 kHz. Each force map consisted of 30 × 30 FDCs (force-distance curves). Young’s modulus (*E*) was calculated from the FDCs of at least 8 measurements per group (*n* ≥ 8) by fitting the DMT model in the AtomicJ software. Significance was determined by ANOVA (*P* < 0.05).

## Supplementary Material

kiag707_Supplementary_Data

## Data Availability

Raw sequencing data with appropriate metadata are available in the NCBI Gene Expression Omnibus (GEO) repository under accession number GSE307383.
