# Supplementary material for: SOBIR1 integrates pathogen recognition and hormonal metabolism in Nicotiana tabacum
Source: Plant Physiol. 2026 Sep 23;202(2):kiag707. doi: 10.1093/plphys/kiag707 (PMC13651904; doi:10.1093/plphys/kiag707)
Supplement: Supplementary file 1 [file kiag707_supplementary_data.zip › Supplementary Figs_Revision2.pdf]

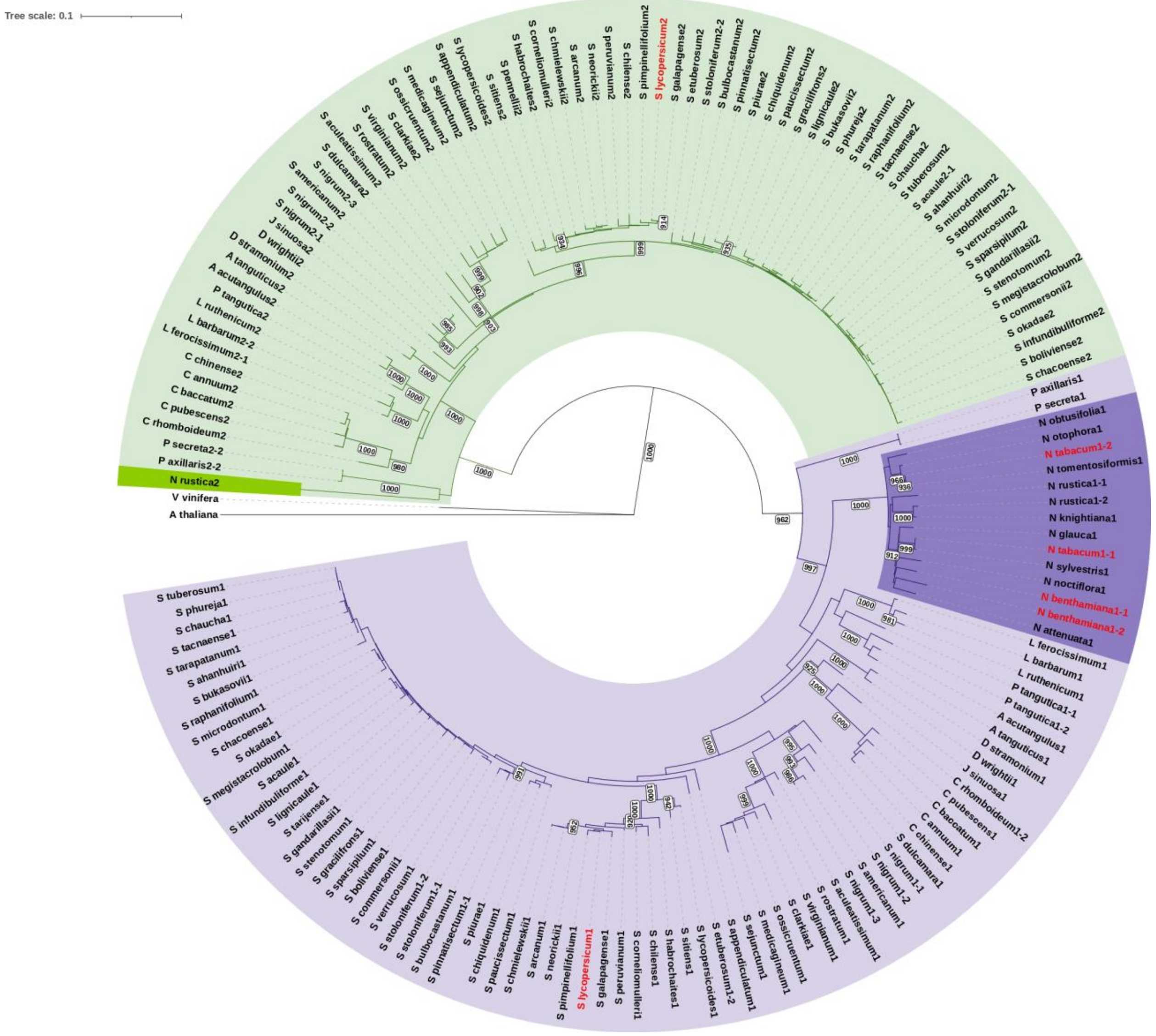

Supplementary Figure S1. Phylogenetic analysis of *SOBIR1* homologs from Solanaceae species*.* 160 sequences were successfully retrieved from the genomes of 81 species. The phylogram was obtained with the fast distance-based phylogeny inference program FastME using 1000 bootstrap replications. The single SOBIR1 from rosid *Vitis vinifera* was used as the outgroup. Two subfamilies or clades were identified. Clade 1 is highlighted in a lilac background and clade 2 in a pale green background. Darker hues highlight SOBIR1 proteins found in Nicotiana genomes. Homologs of interest are marked red. Bootstrap values above 900 are given below the corresponding branch.

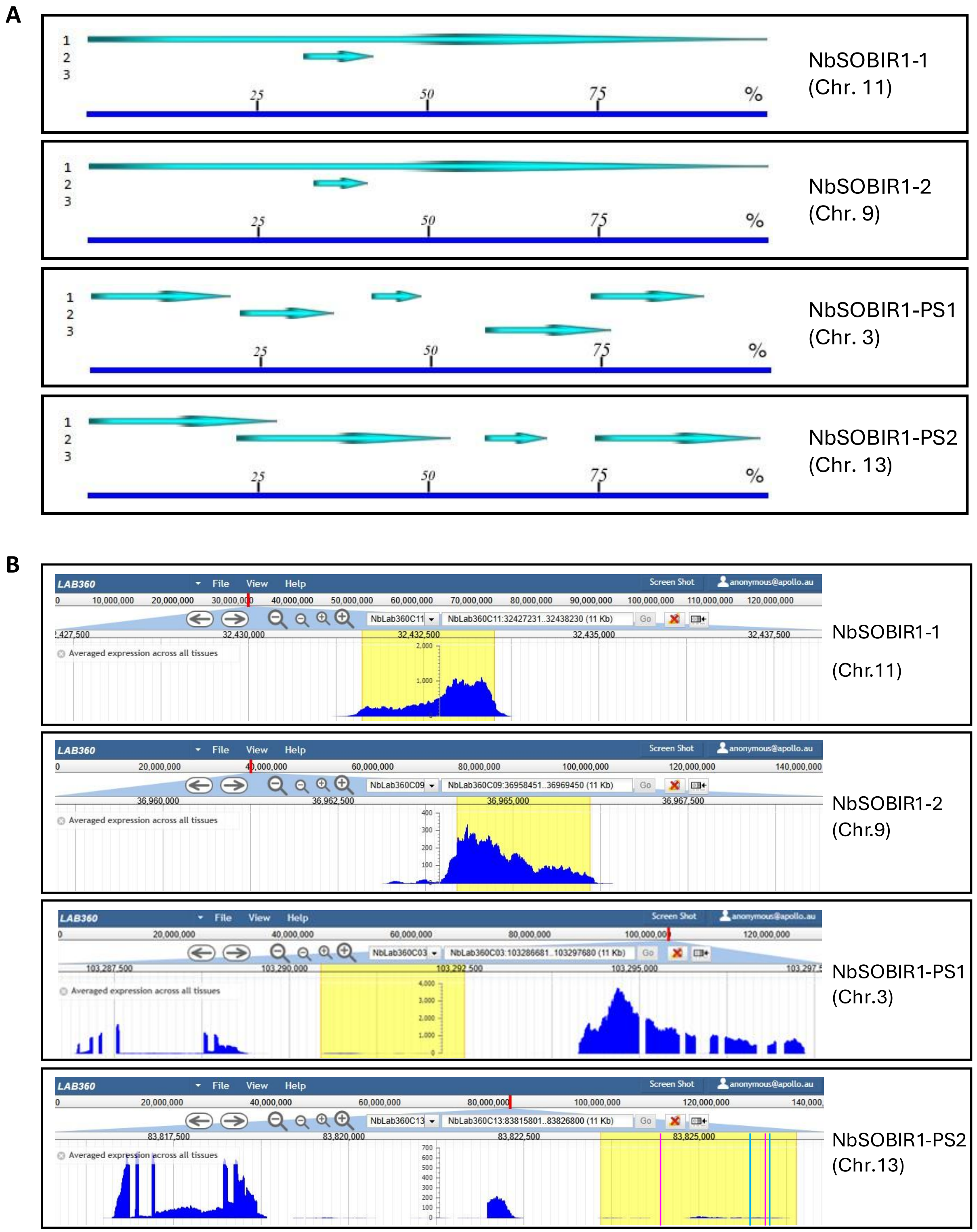

Supplementary Figure S2. Protein-coding potential and expression profiles of *Nicotiana benthamiana* SOBIR1 genes and pseudogenes. (**A**) Open reading frames (ORFs) with a minimum predicted length of 50 amino acids are shown as cyan arrows. Translation was performed using the "DNA to protein translation" tool (Bikandi et al., 2004; http://insilico.ehu.es). (**B**) RNA-seq expression profiles visualized in the *N. benthamiana* Apollo genome browser (https://www.nbenth.com) across 11-kb genomic windows. RNA-seq reads were obtained from total RNA isolated from 10-d-old seedlings and from leaf, flower, stem, and root tissues of 6-week-old plants (Ranawaka, 2024). Gene and pseudogene sequences from start to stop codons are highlighted in yellow. Predicted donor and acceptor splice sites in *NbSOBIR1-PS2* are indicated by blue and pink bars, respectively.

```
NbSOBIR1-1      ATGGCCTTCACTGCTTCACAAATTCACTTCTTTTTCTTCTCACTTTTCGCCTTTTTACTT
NbSOBIR1-2      ATGGCCTTCACTGCTTCACAAATCCACTTCTTTTTCTTCTCCCTTTCCGCCTTTTTACTT
                *********************** ***************** **** *************

NbSOBIR1-1      ATTGCTGTTCAAGCAAGACTGAATCTTTATCCACCAGATCATGCTGCACTTTTGCTTGTC
NbSOBIR1-2      ATTTCTGTTCAAGCAAGACTTAATCTTTATCCACCAGATCATGCTGCACTTTTGCTTGTC
                *** **************** ***************************************

NbSOBIR1-1      CAAAAAGACTTGGGCATCCAAGGTCAACGCATTGC---ACTTTGCAACTCTGCAACAATA
NbSOBIR1-2      CAAAAAGACTTGGGCATCCAAGGTCAACGCATTGCACTACTTTGCAACTCTGCAACAATA
                ***********************************   **********************

NbSOBIR1-1      TCCTGTGAAAGGCGAAAGGCAAACAGAACACAATTGTTGAGAGTCACCCGTATTGACTTC
NbSOBIR1-2      TCCTGTGAAAGGCGAAAGGTAAACAGAACACAATTGTTGAGAGTCACCCGTATTGACTTC
                ******************* ****************************************

NbSOBIR1-1      AGATCCAGTGGATTGAGTGGAACTTTATCTCCTGCCATTGGAAAACTTTCTGTGCTCAAA
NbSOBIR1-2      AGATCCAATGGATTGACTGGAACTTTATCTCCTGCCATTGGAAAACTTTCTGAGCTCAAA
                ******* ******** *********************************** *******

NbSOBIR1-1      GAACTCTCTCTTCCAAACAACCAACTCTTTGACCAAATCCCAGTTCAGATTCTTGATTGC
NbSOBIR1-2      GAGCTCTCTCTTCCAAACAACCAACTCTTTGACCAAATCCCATTTCAGATTCTTCACTGC
                ** *************************************** *********** * ***

NbSOBIR1-1      CGTAAACTGGAGATTCTTGACCTTGGAAACAATCTGTTTTCTGGGAAAGTTCCATCTGAA
NbSOBIR1-2      CGTAAACTGGAGATTCTTGACCTTGGAAACAATCTGTTTTCTGGGAAAGTTCCATCTGAA
                ************************************************************

NbSOBIR1-1      TTATCATCTCTACTCCGCCTTCGAATTCTTGATCTTTCTTCAAATGAGTTTTCTGGGAAT
NbSOBIR1-2      TTATCATCTCTACTCCGCCTTCGAATTCTTGATCTTTCTTCAAATGCGTTTTCTGGGAAT
                ********************************************** *************

NbSOBIR1-1      CTTAACTTCTTGAAGTATTTTCCCAACTTAGAAAAACTCTCTTTAGCTGATAACATGTTC
NbSOBIR1-2      CTTAACTTCTTGAAGTATTTTCCCAACTTAGAGAAACTCTCTTTAGCTGATAACATGTTC
                ******************************** ***************************

NbSOBIR1-1      ACTGGAAAAATACCCCCTTCTTTGAAATCTTTTAGGAATCTCCGTATCCTTAATATTTCA
NbSOBIR1-2      ACTGGAAAAATACCCCCTTCTTTAAAATCTTTTAGGAATCTCCATTTCCTTAATATTTCA
                *********************** ******************* * **************

NbSOBIR1-1      GGAAACAGTTTTCTTGAAGGTCATGTGCCTGTTATGAGTCAAGTTGAGCACTTGTCAGCA
NbSOBIR1-2      GGAAACAGTTTTCTTGAAGGTCCTTTGTCTGTTATGAGTCAAGTTGAGCACTTGTCAGCA
                ********************** * ** ********************************

NbSOBIR1-1      GAATTGGATCAG---CACTTCGTTCCAAAACGTTACATTCTTGCTGAAAATTCAACAAGG
NbSOBIR1-2      GATTTGGATCAGAAAAACTTCGTTCCAAAACGTTACATTCTTGCTGAAAATTCAACAAGG
                ** *********    ********************************************

NbSOBIR1-1      TCAAATCAGATATCAGCACTGGCACCTAATTCCAATTCAGGAAATGCCCCAGCTCCAGCA
NbSOBIR1-2      CCAAATCAGATATCAGCACCGGCACCTAATTCCAATTCAGGAAATGTCCCAGCTCCAGCA
                 ****************** ************************** *************

NbSOBIR1-1      CCGAGTCATAATGTCACTCCAATCCATAAACATAGTAACAGGAAGAAAAGGAAAGTCAGA
NbSOBIR1-2      CCGAGTCATAATGTCGCTTCAATCCATAAACGTAAAAC---AAAAAAAGGAAAGTCAGA
                *************** ** ************ **  ***   ** ***************

NbSOBIR1-1      GCGTGGTTACTTGGTTTCTTTGCTGGTTCTTTCGCAGGAGCTATATCTGCAGTACTCTTA
NbSOBIR1-2      GCGTGGTTTCTTGGTTTCTTTGCTGGTTCTTTCGCAGGGGCTATATCTGCAGTACTCTTA
                ******** ***************************** *********************

NbSOBIR1-1      TCAGTTCTTTTTAAGCTGGTCATGTTTTTTGTCCGAAAGGGAAAGACTGATGGAACTTTA
NbSOBIR1-2      TCAATTCTCTTTAAGCTGGTAATGTTTTTCGTCCGAAAGGGAAAGACTGATGGAACTTTA
                *** **** *********** ******** ******************************

NbSOBIR1-1      ACAATATACAGTCCACTGATTAAGAAAGCCGAGGATTTGGCCTTCTTAGAGACAGAAGAT
NbSOBIR1-2      ACAATATACAGTCCACTGATTAAGAAAGCCGAGGATTTGGCCTTCTTAGAGAACGAAGAT
                ****************************************************  ******

NbSOBIR1-1      GGAGTAGCATCACTTGAAATGATTGGAAAAGGTGGATGCGGAGAAGTTTATAGAGCTGAG
NbSOBIR1-2      GGAGTAGCATCACTTGAATTGATTGGAAAAGGTGGATGTGGAGAAGTTTATAGAGCTGAG
                ****************** ******************* *********************

NbSOBIR1-1      TTACCGGGAAGTAATGGGAAGATTATAGCTATAAAGAAGATTATACAACCCCCAATGGAT
NbSOBIR1-2      TTACCAGGAAGTAATGGGAAAATTATAGCTATAAAGAAGATTATACAACCCCCAATGGAT
                ***** ************** ***************************************

NbSOBIR1-1      GCTGCAGAACTCACCGAGGAAGATACCAAGGCTTTGAACAAAAAAATGCGACAAGTAAAA
NbSOBIR1-2      GCTGCAGAACTCACCGAGGAAGATACCAAGGCTTTGAACAAGAAAATGCGACAGGTAAAA
                ***************************************** *********** ******

NbSOBIR1-1      TCAGAAATTCAAATTCTTGGTCAAATCAGACACAGGAATCTGCTTCCCCTACTGGCACAT
NbSOBIR1-2      TCAGAAATTCAAATTCTTGGTCAAATCAGACACAGGAATCTGCTTCCCCTACTGGCACAT
                ************************************************************

NbSOBIR1-1      ATGCCTAGGCCAGACTGCCATTACTTGGTATATGAATATATGAAAAATGGGAGCTTACAA
NbSOBIR1-2      ATGCCTAGGCCAGACTGCCATTACTTGGTATATGAATATATGAAAAATGGAAGCTTACAA
                ************************************************** *********

NbSOBIR1-1      GATATCCTCCAGCAAGTCACAGAAGGGACAAGGGAATTAGATTGGTTGGGACGACACCGA
NbSOBIR1-2      GATATCCTCCAGCAAGCCACAGAAGGGACAAGGGAATTAGATTGGTTGGGACGACACCGA
                **************** *******************************************

NbSOBIR1-1      ATTGCAGTAGGGATAGCTTCTGGACTTGAGTATCTCCATATAAACCACAGTCAATGCATA
NbSOBIR1-2      ATTGCAGTAGGGATAGCTACTGGACTTGAGTATCTCCATATAAACCACAGTCAATGCATA
                ***************** ******************************************

NbSOBIR1-1      ATTCACAGAGATCTAAAGCCAGCAAATGTCCTTCTTGACGATGATATGGAAGCTCGAATT
NbSOBIR1-2      ATTCACAGAGATCTAAAGCCAGCAAATGTCCTTCTTGACGATGATATGGAAGCTCGAATA
                ***********************************************************

NbSOBIR1-1      GCTGATTTTGGACTTGCAAAAGCACTCCCAGATGCCCATACACATGTTACGACTTCAAAT
NbSOBIR1-2      GCTGATTTTGGACTTGCAAAGGCACTTCCAGATGCCCATACACATGTTACGACTTCAAAT
                ******************** ***** *********************************

NbSOBIR1-1      GTTGCAGGAACTGTGGGATATATTGCACCAGAATACCATCAGACACTGAAGTTTACGGGT
NbSOBIR1-2      GTTGCAGGAACTGTGGGATATATTGCACCAGAATACCATCAGACACTGAAGTTTACGGAT
                ********************************************************** *

NbSOBIR1-1      AAGTGTGATATATACAGCTTTGGTGTGGTGTTGGCAGTGCTGGTTATAGGAAAACTTCCA
NbSOBIR1-2      AAGTGTGATATATACAGCTTTGGTGTGGTGTTAGCAGTGCTGGTTATAGGAAAGCTTCCA
                ******************************** ******************** ******

NbSOBIR1-1      TCAGATGAATTTTTCCAGCACACGCCTGAGATGAGTTTAGTGAAGTGGCTGAGAAATGTA
NbSOBIR1-2      TCAGATGAATTTTTCCAGCACACGCCTGAGATGAGTTTAGTGAAGTGGCTGAGAAATGTA
                ************************************************************

NbSOBIR1-1      ATGACTTCTGAGGATCCGAAAAGGGCAATTGATTCAAAGCTGATAGGAAATGGATTTGAG
NbSOBIR1-2      ATGACTTCTGAGGATCCGAAAAAGGCAATTGATCCAAAGCTAATAGGAAATGGATTTGAG
                ********************** ********** ******* ******************

NbSOBIR1-1      GAGCAAATGCTTTTGGTTCTCAAGATAGCTTGCTTTTGTACTCTGGAAAATCCAAAGGAG
NbSOBIR1-2      GAGCAAATGCTTTTGGTTCTCAAGATAGCTTGCTTTTGTACTCTGGAGAATCCAAAGGAG
                *********************************************** ************

NbSOBIR1-1      AGGCCTAACAGTAAGGATGTTAGGTGTATGTTAACTCAGATCAAGCATTAA
NbSOBIR1-2      AGGCCTAACAGTAAGGATGTTAGGTGTATGTTAACTCAGATCAAGCATTAG
                **************************************************
```

Supplementary Figure S3. Sequence alignment of genes *NbSOBIR1-1* and *NbSOBIR1-2*. The aligned sequences extend from the predicted start and stop codons of their single ORF. The sequences were aligned using MUSCLE software (Madeira F, Madhusoodanan N, Lee J, Eusebi A, Niewielska A, Tivey ARN, Lopez R, Butcher S. The EMBL-EBI Job Dispatcher sequence analysis tools framework in 2024. Nucleic Acids Res. 2024 Jul 5;52(W1): W521-W525. doi: 10.1093/nar/gkae241).

```
NbSOBIR1-like-LH      ATGGCCTCCACTACCTCCCATATTCACCTATCTCTTCTGTCTCTCTTCACTCTTATCCTT
NbSOBIR1-PS1          ATGGCCTCCACTACCTCCCATATTCACCTATCTCTTCTGTCTCTCTTCACTCTTATCCTT
                      ************************************************************

NbSOBIR1-like-LH      CATGTTCAAGCAAGAATCAACCTTTACTCGCCAGATTATAGTGCTCTTTTGGTTGTCCGA
NbSOBIR1-PS1          CATGTTCAAGCAAGAATCAACCTTTACTCGCCAGATTATAGTGCTCTTTTGGTTGTCCGA
                      ************************************************************

NbSOBIR1-like-LH      AAAGGCTTAGACGTCCCTAGACAACTTAGTGCTATAGAGAACCCATGCAATTCTGTTGGA
NbSOBIR1-PS1          AAAGGCTTAGACGTCCCTAGACAACTTAGTGCTATAGAGAACCCATGCAATTCTGTTGGA
                      ************************************************************

NbSOBIR1-like-LH      ATATCATGTGAACGACGACTCACAAACAATTCATATGTGCTTAGAGTCACAGGGGTTGTT
NbSOBIR1-PS1          ATATCATGTGAACGACGACTCACAAACAATTCATATGTGCTTAGAGTCACAGGGGTTGTT
                      ************************************************************

NbSOBIR1-like-LH      TTCAAGTCCTATGAACTGAGGGCAACTCTATCTCCTGCCATTTGCAAGCTTTCTGAGTTC
NbSOBIR1-PS1          TTCAAGTCCTATGAACTGAGGGCAACTCTATCTCCTGCCATTTGCAAGCTTTCTGAGTTC
                      ************************************************************

NbSOBIR1-like-LH      AAAAAATTGTCCCTACAAAACAACAAGCTCTTTGACAGAATCCCAACTCAAATTGTTGAG
NbSOBIR1-PS1          AAAAAATTGTCCCTACAAAACAACAAGCTCTTTGACAGAATCCCAACTCAAATTGTTGAG
                      ************************************************************

NbSOBIR1-like-LH      GTGCCGAAAATTGGAAATCTTGAACCTTCAAAACAACCAGTTTTCTGGCAAAGTCCCATC
NbSOBIR1-PS1          GTGCCGAAAATTGGAAATCTTGAACCTTCAAAACAACCAGTTTTCTGGCAAAGTCCCATC
                      ************************************************************

NbSOBIR1-like-LH      TGAATTATCATCTCTTGTCCGCCTTCGAATCCTTGACCTCTCTTCTAATGAATTATCAGG
NbSOBIR1-PS1          TGAATTATCATCTCTTGTCCGCCTTCGAATCCTTGACCTCTCTTCTAATGAATTATCAGG
                      ************************************************************

NbSOBIR1-like-LH      GAACCTCAATTTCTTGAAATACTTTCCCAACCTTGAAAATTTGTCCCTTGCTGATAACAT
NbSOBIR1-PS1          GAACCTCAATTTCTTGAAATACTTTCCCAACCTTGAAAATTTGTCCCTTGCTGATAACAT
                      ************************************************************

NbSOBIR1-like-LH      GTTCATTGACAAAATACCTCAATCCTTGAAATCTTTCAGAAATCTCCGGTTCCTCAACAT
NbSOBIR1-PS1          GTTCATTGACAAAATACCTCAATCCTTGAAATCTTTCAGAAATCTCCGGTTCCTCAACAT
                      ************************************************************

NbSOBIR1-like-LH      TTCAGGGAATAGTTTTCTTTAAGGTCCGGTGCCTTTCATGAGTCAAGTTAAGCACTTATC
NbSOBIR1-PS1          TTCAGGGAATAGTTTTCTTTAAGGTCCGGTGCCTTTCATGAGTCAAGTTAAGCACTTATC
                      ************************************************************

NbSOBIR1-like-LH      AGCAGACTTGAATCGAAAAGATTATGTTCTTAAACGTTACATTCTTGCTGAAAACTCAAC
NbSOBIR1-PS1          AGCAGACTTGAATCGAAAAGATTATGTTCTTAAACGTTACATTCTTGCTGAAAACTCAAC
                      ************************************************************

NbSOBIR1-like-LH      AAGGTCAAGTCACAAATCTGCAATGGCACCACAGTCCTATTCAGGAAATGCCCCGGCTCC
NbSOBIR1-PS1          AAGGTCAAGTCACAAATCTGCAATGGCACCACAGTCCTATTCAGGAAATGCCCCGGCTCC
                      ************************************************************

NbSOBIR1-like-LH      AGGACCCAGTGCAATTGTAGTACCAGTACACAAACATAACAACACAAAAAAGAAGGTAAG
NbSOBIR1-PS1          AGGACCCAGTGCAATTGTAGTACCAGTACACAAACATAACAACACAAAAAAGAAGGTAAG
                      ************************************************************

NbSOBIR1-like-LH      CATATGGATTCTTGGATTCCTGGCTGGATCTTTTGCAGGGATCTTATCTGCGTGGTCTTA
NbSOBIR1-PS1          CATATGGATTCTTGGATTCCTGGCTGGATCTTTTGCAGGGATCTTATCTGCGTGGTCTTA
                      ************************************************************

NbSOBIR1-like-LH      TCCTTGCTCTTCAAGGTGGTTATGATTATCGGAGGGAGCAAGGATGATTCAGGTATAACA
NbSOBIR1-PS1          TCCTTGCTCTTCAAGGTGGTTATGATTATCGGAGGGAGCAAGGATGATTCAGGTATAACA
                      ************************************************************

NbSOBIR1-like-LH      ATTTTCAGTCCATTGATTAAG-----------------------AAAGCAGAGTTACCAG
NbSOBIR1-PS1          ATTTTCAGTCCATTGATTAAGAAGGTGGATGTGGATTAGTTTATAAAGCAGAGTTACCAG
                      *********************                       ****************

NbSOBIR1-like-LH      GAAGTAATGGAAAGATTATTGCTATAAAGAAGATCGTAGAACCCCCAAAGGATGCTGCAG
NbSOBIR1-PS1          GAAGTAATGGAAAGATTATTGCTATAAAGAAGATCGTAGAACCCCCAAAGGATGCTGCAG
                      ************************************************************

NbSOBIR1-like-LH      AA----------------------------------------------------------
NbSOBIR1-PS1          AACTCACTGAGGAAGATAGCAACAACAACAACCCAGTATAATCCTATTTAGTAAGGTCTG
                      **

NbSOBIR1-like-LH      ------------------------------------------------------------
NbSOBIR1-PS1          GGGAAGGTATTGTGTACGCATACCTTACCCCTACCCTTGGGTAGAGAGGCTGTTTCCAAA

NbSOBIR1-like-LH      ------------------------------------------------------------
NbSOBIR1-PS1          TAGACCCCCAGCATCCTTCCCTCCAAGAACTTCCCACCTTGCTCTTCGGGAGATTCGAAC

NbSOBIR1-like-LH      --------------------------------------------------------CTCA
NbSOBIR1-PS1          GCACAACCTCTCGGTTGGAAGTGGGGGTTGCTTACCATCAGAGCAACCCCTCTTGTCTCA
                                                                              ****

NbSOBIR1-like-LH      CTGAGGAAGATAGCAAGGCTTTGAATATTAAAATGTGCCAGACCAAATCAGAAATTAAAA
NbSOBIR1-PS1          CTGAGGAAGATAGCAAGGCTTTGAATATTAAAATGTGCCAGACCAAATCAGAAATTAAAA
                      ************************************************************

NbSOBIR1-like-LH      TTGTAGGTCAAATAAGACACAGGAATTTGCTTCCTCTAATAGCACATATGCCAAGACCTG
NbSOBIR1-PS1          TTGTAGGTCAAATAAGACACAGGAATTTGCTTCCTCTAATAGCACATATGCCAAGACCTG
                      ************************************************************

NbSOBIR1-like-LH      ACTGCCATTACTTGGTCCGTGAGTACATGAAAAATGGGAGCTTACAGGATGCCGTCCAGC
NbSOBIR1-PS1          ACTGCCATTACTTGGTCCGTGAGTACATGAAAAATGGGAGCTTACAGGATGCCGTCCAGC
                      ************************************************************

NbSOBIR1-like-LH      AAGTCACGGAGGGGACACGGGAAATAGATTGGTCGGCACGTCATCGAATTGCAGTCGGGA
NbSOBIR1-PS1          AAGTCACGGAGGGGACACGGGAAATAGATTGGTCGGCACGTCATCGAATTGCAGTCGGGA
                      ************************************************************

NbSOBIR1-like-LH      TCGCTGCTGGGCTTGAGTATCTTCATGTAAATCATAGTCAGCGTACAATTCACAGGGATC
NbSOBIR1-PS1          TCGCTGCTGGGCTTGAGTATCTTCATGTAAATCATAGTCAGCGTACAATTCACAGGGATC
                      ************************************************************

NbSOBIR1-like-LH      TAAAGCCAGCAAATGTCCTTCTTGCGAAAGCAATCCCAGAATCCCTTACGCTTTTCAACT
NbSOBIR1-PS1          TAAAGCCAGCAAATGTCCTTCTTGCGAAAGCAATCCCAGAATCCCTTACGCTTTTCAACT
                      ************************************************************

NbSOBIR1-like-LH      TCACACGTGGTAGGAACTTTAGGATACATTGCACCAGAATATTACCAGACCGTTAAGTTC
NbSOBIR1-PS1          TCACACGTGGTAGGAACTTTAGGATACATTGCACCAGAATATTACCAGACCGTTAAGTTC
                      ************************************************************

NbSOBIR1-like-LH      ACAGATAAGTGTGATATATACAGCTTCGGGATGCTGCTAGGCGTGCTAGTTATGGGAAAG
NbSOBIR1-PS1          ACAGATAAGTGTGATATATACAGCTTCGGGATGCTGCTAGGCGTGCTAGTTATGGGAAAG
                      ************************************************************

NbSOBIR1-like-LH      TTTCCCTCTGATGAGCTCTTCCAGCCGGTTTCTGGGATGGGTTTAGTGAAATGGATGAGA
NbSOBIR1-PS1          TTTCCCTCTGATGAGCTCTTCCAGCCGGTTTCTGGGATGGGTTTAGTGAAATGGATGAGA
                      ************************************************************

NbSOBIR1-like-LH      AATGTCACGACTTCTGAGAATCCAAAAAGAGCAATTGATCCAAAGCTGATGGGTAATGGG
NbSOBIR1-PS1          AATGTCACGACTTCTGAGAATCCAAAAAGAGCAATTGATCCAAAGCTGATGGGTAATGGG
                      ************************************************************

NbSOBIR1-like-LH      TATGAGGAGCAAAATGCTTTTGGTTCTCAAGATTGCCTGCTTTTGCTCACTGGATAATGC
NbSOBIR1-PS1          TATGAGGAGCAAAATGCTTTTGGTTCTCAAGATTGCCTGCTTTTGCTCACTGGATAATGC
                      ************************************************************

NbSOBIR1-like-LH      AAAGGAGAGGCCTAACAGTAAGGATGTTAGATGCATGTTAACTCAGATCAAGCCTCAAGT
NbSOBIR1-PS1          AAAGGAGAGGCCTAACAGTAAGGATGTTAGATGCATGTTAACTCAGATCAAGCCTCAAGT
                      ************************************************************

NbSOBIR1-like-LH      AGGTAGAGGATAA
NbSOBIR1-PS1          AGGTAGAGGATAA
                      *************
```

Supplementary Figure S4. Sequence alignment of *SOBIR1-like* gene described by Liebrand et al. (2013) and Huang et al. (2021) (NbSOBIR1-like-LH) and the corresponding sequence found in the *N. benthamiana* reference genome (NbSOBIR1-PS1). The aligned sequences extend from putative start and stop codons as deduced from the alignment with NbSOBIR1 genes. The SINE element Nt3 is italicized. The target site duplication resulting from the insertion is underlined.

```
NbSOBIR1-1      ATGGCCTTCACTGCTTCACAAATTCACTTCTTTTTCTTCTCACTTTTCGCCTTTTTACTT
NbSOBIR1-PS1    ATGGCCTCCACTACCTCCCATATTCACCTATCTCTTCTGTCTCTCTTCACTCTTATCCTT
                ******* **** * ** ** ****** * * * *  * ** ** *** *  ** * ***

NbSOBIR1-1      ATTGCTGTTCAAGCAAGACTGAATCTTTATCCACCAGATCATGCTGCACTTTTGCTTGTC
NbSOBIR1-PS1    C---ATGTTCAAGCAAGAATCAACCTTTACTCGCCAGATTATAGTGCTCTTTTGGTTGTC
                     ************* * ** *****  * ****** **  *** ****** *****

NbSOBIR1-1      CAAAAAGACTTGGGCATCCAAGGTCAACGCATTGC---------ACTTTGCAACTCTGCA
NbSOBIR1-PS1    CGAAAAGGCTTAGACGTCCCTAGACAACTTAGTGCTATAGAGAACCCATGCAATTCTGTT
                * ***** *** * * ***   * ****  * ***          *  ***** ****

NbSOBIR1-1      ACAATATCCTGTGAAAGGCGAAAGGCAAACAGAACACAATTGTTGAGAGTCACCCGTATT
NbSOBIR1-PS1    GGAATATCATGTGAACGACGACTCACAAACAATTCATATGTGCTTAGAGTCACAGGGGTT
                  ****** ****** * ***    ******   ** *  ** * ********  *  **

NbSOBIR1-1      GACTTCAGATCCAGTGGATTGAGTGGAACTTTATCTCCTGCCATTGGAAAACTTTCTGTG
NbSOBIR1-PS1    GTTTTCAAGTCCTATGAACTGAGGGCAACTCTATCTCCTGCCATTTGCAAGCTTTCTGAG
                *  ****  ***  ** * **** * **** ************** * ** ******* *

NbSOBIR1-1      CTCAAAGAACTCTCTCTTCCAAACAACCAACTCTTTGACCAAATCCCAGTTCAGATTCTT
NbSOBIR1-PS1    TTCAAAAAATTGTCCCTACAAAACAACAAGCTCTTTGACAGAATCCCAACTCAAATTGTT
                 ***** ** * ** ** * ******* * *********  *******  *** *** **

NbSOBIR1-1      GA-TTGCCGTAAACTGGAGATTCTTGACCTTGGAAACAATCTGTTTTCTGGGAAAGTTCC
NbSOBIR1-PS1    GAGGTGCCGAAAATTGGAAATCTTGAACCTTCAAAACAACCAGTTTTCTGGCAAAGTCCC
                **  ***** *** **** **  *  *****  ****** * ********* ***** **

NbSOBIR1-1      ATCTGAATTATCATCTCTACTCCGCCTTCGAATTCTTGATCTTTCTTCAAATGAGTTTTC
NbSOBIR1-PS1    ATCTGAATTATCATCTCTTGTCCGCCTTCGAATCCTTGACCTCTCTTCTAATGAATTATC
                ******************  ************* ***** ** ***** ***** ** **

NbSOBIR1-1      TGGGAATCTTAACTTCTTGAAGTATTTTCCCAACTTAGAAAAACTCTCTTTAGCTGATAA
NbSOBIR1-PS1    AGGGAACCTCAATTTCTTGAAATACTTTCCCAACCTTGAAAATTTGTCCCTTGCTGATAA
                 ***** ** ** ******** ** ********* * *****  * **  * ********

NbSOBIR1-1      CATGTTCACTGGAAAAATACCCCCTTCTTTGAAATCTTTTAGGAATCTCCGTATCCTTAA
NbSOBIR1-PS1    CATGTTCATTGACAAAATACCTCAATCCTTGAAATCTTTCAGAAATCTCCGGTTCCTCAA
                ******** **  ******** *  ** *********** ** ********  **** **

NbSOBIR1-1      TATTTCAGGAAACAGTTTTCTTGAAGGTCATGTGCCTGTTATGAGTCAAGTTGAGCACTT
NbSOBIR1-PS1    CATTTCAGGGAATAGTTTTCTTTAAGGTCCGGTGCCTTTCATGAGTCAAGTTAAGCACTT
                 ******** ** ********* ******  ****** * ************ *******

NbSOBIR1-1      GTCAGCAGAATTGGATC---AGCACTTCGTTCCAAAACGTTACATTCTTGCTGAAAATTC
NbSOBIR1-PS1    ATCAGCAGACTTGAATCGAAAAGATTATGTTCTTAAACGTTACATTCTTGCTGAAAACTC
                 ******** *** ***   *  * *  ****  ********************** **

NbSOBIR1-1      AACAAGGTCAAATCAGATATCAGCACTGGCACCTAATTCCAATTCAGGAAATGCCCCAGC
NbSOBIR1-PS1    AACAAGGTCAAGTCACAAATCTGCAATGGCACCACAGTCCTATTCAGGAAATGCCCCGGC
                *********** *** * *** *** *******  * *** **************** **

NbSOBIR1-1      TCCAGCACCGAGTCATAATGTCACTCCAATCCATAAACATAGTAACAGGAAGAAAAGGAA
NbSOBIR1-PS1    TCCAGGACCCAGTGCAATTGTAGTACCAGTACACAAACATAACAAC---ACAAAAAAGAA
                ***** *** ***   * ***    *** * ** *******  ***   *  **** ***

NbSOBIR1-1      AGTCAGAGCGTGGTTACTTGGTTTCTTTGCTGGTTCTTTCGCAGGAGCTATATCTGCAGT
NbSOBIR1-PS1    GGTAAGCATATGGATTCTTGGATTCCTGGCTGGATCTTTTGCAGGGATCTTATCTGC-GT
                 ** **    *** * ***** *** * ***** ***** *****     ******* **

NbSOBIR1-1      ACTCTTATCAGTTCTTTTTAAGCTGGTCATGTTTTTTGTCCGAAAGGGAAAGACTGATGG
NbSOBIR1-PS1    GGTCTTATCCTTGCTCTTCAAGGTGGTTATGATTATCG---GAGGGAGCAAGGATGATTC
                  *******  * ** ** *** **** *** ** * *   **  * * ***  ****

NbSOBIR1-1      AACTTTAACAATATACAGTCCACTGATTAAGAAAGCCGAGGATTTGGCCTTCTTAGAGAC
NbSOBIR1-PS1    AGGTATAACAATTTTCAGTCCATTGATTAAG-----------------------------
                *  * ******* * ******* ********

NbSOBIR1-1      AGAAGATGGAGTAGCATCACTTGAAATGATTGGAAAAGGTGGATGCGGAGAAGTTTATAG
NbSOBIR1-PS1    -----------------------------------AAGGTGGATGTGGATTAGTTTATAA
                                                   ********** ***  ********

NbSOBIR1-1      AGCTGAGTTACCGGGAAGTAATGGGAAGATTATAGCTATAAAGAAGATTATACAACCCCC
NbSOBIR1-PS1    AGCAGAGTTACCAGGAAGTAATGGAAAGATTATTGCTATAAAGAAGATCGTAGAACCCCC
                *** ******** *********** ******** *************  ** *******

NbSOBIR1-1      AATGGATGCTGCAGAA--------------------------------------------
NbSOBIR1-PS1    AAAGGATGCTGCAGAACTCACTGAGGAAGATAGCAACAACAACAACCCAGTATAATCCTA
                ** *************

NbSOBIR1-1      ------------------------------------------------------------
NbSOBIR1-PS1    TTTAGTAAGGTCTGGGGAAGGTATTGTGTACGCATACCTTACCCCTACCCTTGGGTAGAG

NbSOBIR1-1      ------------------------------------------------------------
NbSOBIR1-PS1    AGGCTGTTTCCAAATAGACCCCCAGCATCCTTCCCTCCAAGAACTTCCCACCTTGCTCTT

NbSOBIR1-1      ------------------------------------------------------------
NbSOBIR1-PS1    CGGGAGATTCGAACGCACAACCTCTCGGTTGGAAGTGGGGGTTGCTTACCATCAGAGCAA

NbSOBIR1-1      ----------CTCACCGAGGAAGATACCAAGGCTTTGAACAAAAAAATGCGACAAGTAAA
NbSOBIR1-PS1    CCCCTCTTGTCTCACTGAGGAAGATAGCAAGGCTTTGAATATTAAAATGTGCCAGACCAA
                          ***** ********** ************ *  ****** * **    **

NbSOBIR1-1      ATCAGAAATTCAAATTCTTGGTCAAATCAGACACAGGAATCTGCTTCCCCTACTGGCACA
NbSOBIR1-PS1    ATCAGAAATTAAAATTGTAGGTCAAATAAGACACAGGAATTTGCTTCCTCTAATAGCACA
                ********** ***** * ******** ************ ******* *** * *****

NbSOBIR1-1      TATGCCTAGGCCAGACTGCCATTACTTGGTATATGAATATATGAAAAATGGGAGCTTACA
NbSOBIR1-PS1    TATGCCAAGACCTGACTGCCATTACTTGGTCCGTGAGTACATGAAAAATGGGAGCTTACA
                ****** ** ** *****************   *** ** ********************

NbSOBIR1-1      AGATATCCTCCAGCAAGTCACAGAAGGGACAAGGGAATTAGATTGGTTGGGACGACACCG
NbSOBIR1-PS1    GGATGCCGTCCAGCAAGTCACGGAGGGGACACGGGAAATAGATTGGTCGGCACGTCATCG
                 ***  * ************* ** ****** ***** ********* ** *** ** **

NbSOBIR1-1      AATTGCAGTAGGGATAGCTTCTGGACTTGAGTATCTCCATATAAACCACAGTCAATGCAT
NbSOBIR1-PS1    AATTGCAGTCGGGATCGCTGCTGGGCTTGAGTATCTTCATGTAAATCATAGTCAGCGTAC
                ********* ***** *** **** *********** *** **** ** *****  * *

NbSOBIR1-1      AATTCACAGAGATCTAAAGCCAGCAAATGTCCTTCTTGACGATGATATGGAAGCTCGAAT
NbSOBIR1-PS1    AATTCACAGGGATCTAAAGCCAGCAAATGTCCTT--------------------------
                ********* ************************

NbSOBIR1-1      TGCTGATTTTGGACTTGCAAAAGCACTCCCAGATGCCCATACACATGTTACGACTTCAAA
NbSOBIR1-PS1    ------------CTTGCGAAAGCAATCCCAGAATCCCTTACGC--TTTTCAACTTCACA
                            ***** ****** *******  *** *** *   ** * ****** *

NbSOBIR1-1      TGTTGCAGGAACTGTGGGATATATTGCACCAGAATACCATCAGACACTGAAGTTTACGGG
NbSOBIR1-PS1    CGTGGTAGGAACTTTAGGATACATTGCACCAGAATATTACCAGACCGTTAAGTTCACAGA
                 ** * ******* * ***** **************  * *****  * ***** ** *

NbSOBIR1-1      TAAGTGTGATATATACAGCTTTGGTGTGGTGTTGGCAGTGCTGGTTATAGGAAAACTTCC
NbSOBIR1-PS1    TAAGTGTGATATATACAGCTTCGGGATGCTGCTAGGCGTGCTAGTTATGGGAAAGTTTCC
                ******************** **  ** ** * *  ***** ***** *****  ****

NbSOBIR1-1      ATCAGATGAATTTTTCCAGCACACGCCTGAGATGAGTTTAGTGAAGTGGCTGAGAAATGT
NbSOBIR1-PS1    CTCTGATGAGCTCTTCCAGCCGGTTTCTGGGATGGGTTTAGTGAAATGGATGAGAAATGT
                 ** *****  * *******      *** **** ********** *** **********

NbSOBIR1-1      AATGACTTCTGAGGATCCGAAAAGGGCAATTGATTCAAAGCTGATAGGAAATGGATTTGA
NbSOBIR1-PS1    CACGACTTCTGAGAATCCAAAAAGAGCAATTGATCCAAAGCTGATGGGTAATGGGTATGA
                 * ********** **** ***** ********* ********** ** ***** * ***

NbSOBIR1-1      GGAGC-AAATGCTTTTGGTTCTCAAGATAGCTTGCTTTTGTACTCTGGAAAATCCAAAGG
NbSOBIR1-PS1    GGAGCAAAATGCTTTTGGTTCTCAAGATTGCCTGCTTTTGCTCACTGGATAATGCAAAGG
                ***** ******************** ** ********  * ***** *** ******

NbSOBIR1-1      AGAGGCCTAACAGTAAGGATGTTAGGTGTATGTTAACTCAGATCAAGCATTAA-------
NbSOBIR1-PS1    AGAGGCCTAACAGTAAGGATGTTAGATGCATGTTAACTCAGATCAAGCCTCAAGTAGGTA
                ************************ ** ****************** * **

NbSOBIR1-1      --------
NbSOBIR1-PS1    GAGGATAA
```

Supplementary Figure S5: Sequence alignment of gene *NbSOBIR1-1* and pseudogene *NbSOBIR1-PS1*. The aligned sequences extend from the start and stop codons of NbSOBIR1-1 single ORF. The SINE element Nt3 is italicized. The target site duplication resulting from the insertion is underlined.

```
NbSOBIR1-PS2        ATGTCTTTCTTCACTCTTATCCTTGTTGTTCAAGCAAGACTCAACCTTTACTCGCCAGAT
NbSOBIR1L-cDNA-P    ATGTCTTTCTTCACTCTTATCCTTGTTGTTCAAGCAAGACTCAACCTTTACTCGCCAGAT
                    ************************************************************

NbSOBIR1-PS2        TATAGTGCTCTTTTGCTTGTCCAAAAAGGCTTAAACGTCCCTGGACAACGTAGTGCTATA
NbSOBIR1L-cDNA-P    TATAGTGCTCTTTTGCTTGTCCAAAAAGGCTTAAACGTCCCTGGACAACGTAGTGCTATA
                    ************************************************************

NbSOBIR1-PS2        GAGAACCCATGCAACTCTGTTGGAATATCATGTGAACGACAATTCACAAACAATTCATAC
NbSOBIR1L-cDNA-P    GAGAACCCATGCAACTCTGTTGGAATATCATGTGAACGACAATTCACAAACAATTCATAC
                    ************************************************************

NbSOBIR1-PS2        GTGCTTAGAGTCACAGGGATCGTTTTCAAGTCCTATGAACTGAGGGGAACTCTATCTCCT
NbSOBIR1L-cDNA-P    GTGCTTAGAGTCACAGGGATCGTTTTCAAGTCCTATGAACTGAGGGGAACTCTATCTCCT
                    ************************************************************

NbSOBIR1-PS2        GCTATTGGCAAGCTTTCGGAGCTCAAAGTGTTGTCCCTCCAAAACAACCTCTTTGACAGA
NbSOBIR1L-cDNA-P    GCTATTGGCAAGCTTTCGGAGCTCAAAGTGTTGTCCCTCCAAAACAACCTCTTTGACAGA
                    ************************************************************

NbSOBIR1-PS2        ATCCCAACTCAAATTGTTGA-ATGTCGAAAATTGGAAATCTTGAACCTTCAAAACAACCA
NbSOBIR1L-cDNA-P    ATCCCAACTCAAATTGTTGAGGTGCCGAAAATTGGAAATCTTGAACCTTCAAAACAACCA
                    ********************  ** ***********************************

NbSOBIR1-PS2        GTTTTCTGGCAAAGTCCCATCTGAATTATCATCTCTTGTCCGCCTTCGAATCCTTGACCT
NbSOBIR1L-cDNA-P    GTTTTCTGGCAAA-----------------------------------------------
                    *************

NbSOBIR1-PS2        CTCTTATAATGAGTTATCAGGGAACCTCAATTTCTTGAAATACTTCCCCAACTTTGAAAA
NbSOBIR1L-cDNA-P    --------------------GGAACCTCAATTTCTTGAAATACTTCCCCAACTTTGAAAA
                                        ****************************************

NbSOBIR1-PS2        ATTGTCCCTTGGTGATAACATGTTCACTAGCAAAATACCTCAATCCTTGAAATCTTTCAG
NbSOBIR1L-cDNA-P    ATTGTCCCTTGGTGATAACATGTTCACTAGCAAAATACCTCAATCCTTGAAATCTTTCAG
                    ************************************************************

NbSOBIR1-PS2        AAATCCCCGGTTCCTCAACATTTCAGGGAATAGTTTCCTTGAAGGTCCGGTGCCTTTCAC
NbSOBIR1L-cDNA-P    AAATCCCCGGTTCCTCAACATTTCAGGGAATAGTTTCCTTGAAGGTCCGGTGCCTTTCAC
                    ************************************************************

NbSOBIR1-PS2        GAGTCAAGTTGAGCACTTATCAGCAGACTTGAATCGAAAAGATTATGTTCTTAAGCGTTA
NbSOBIR1L-cDNA-P    GAGTCAAGTTGAGCACTTATCAGCAGACTTGAATCGAAAAGATTATGTTCTTAAGCGTTA
                    ************************************************************

NbSOBIR1-PS2        CATTCTTGCTGAAAACTCAACAAGGTCAAGTCACAAATCTGCAATGGCACCAGTCCTATT
NbSOBIR1L-cDNA-P    CATTCTTGCTGAAAACTCAACAAG------------------------------------
                    ************************

NbSOBIR1-PS2        CAGGAAATGCCCCGGCTCCAGGACCAAGTGCAATTGTAGTACCAGCACACAAACATAACA
NbSOBIR1L-cDNA-P    ------------------------------------------------------------

NbSOBIR1-PS2        CCACAAAAAAGAAGGTAAGCATATGGATTCTTGGATTCCGGGCTGGATCTTTTGCAGGGG
NbSOBIR1L-cDNA-P    ------------------------------------------------------------

NbSOBIR1-PS2        TCTTATCTGCGGTGGTCTTATCCTTGCTCTTCAAGGTGGTTATGATTTTTATCAGAGGGA
NbSOBIR1L-cDNA-P    ------------------------------------------------------------

NbSOBIR1-PS2        GCAAGGATGATTCAGGTGTAACAGTTTTCAGTCCATTGATTAAGAAAGCCGAGGACTTGA
NbSOBIR1L-cDNA-P    ------------------------------------------------------------

NbSOBIR1-PS2        CATTTTTAGAGAACGTAGACGGATTGGCGGGGGATGGAGAGATTGACGGGGACGTCACCC
NbSOBIR1L-cDNA-P    ------------------------------------------------------------

NbSOBIR1-PS2        ATCGGATAGGAGCGGGGTGGTCAAAGTGGAGGCTTGCTTCAGGGGTCCTGTGTGACAAGA
NbSOBIR1L-cDNA-P    ------------------------------------------------------------

NbSOBIR1-PS2        AAATTCTACACAAGCTCAAGGGGAAATTCTACAGAGCTGTGGTTAGACCTGCGATGTTAT
NbSOBIR1L-cDNA-P    ------------------------------------------------------------

NbSOBIR1-PS2        ATGGGGCGGAGTGCTGGCCCGTGAAGAACTCGCATGTTCAGAGGATGAAGGTTGCAGAGA
NbSOBIR1L-cDNA-P    ------------------------------------------------------------

NbSOBIR1-PS2        TGAGGATGATTAGGTGGATGTGTGGGCACACCAGGCTGGATAAGATCAAGAATGAAGTTA
NbSOBIR1L-cDNA-P    ------------------------------------------------------------

NbSOBIR1-PS2        TTCGTCAGAAAGTAGGGGTAGCCCCGGTGGACGAGAAGATGAGAGAAGCGAGGCTGAGGT
NbSOBIR1L-cDNA-P    ------------------------------------------------------------

NbSOBIR1-PS2        GGTTCGGGCATGTTCGGAGGAGGGCCCCCGATGCCCCGGTAAGGAGCTGCGAGCGGCTGG
NbSOBIR1L-cDNA-P    ------------------------------------------------------------

NbSOBIR1-PS2        AGATTGTAGGTGGTCGGCGAGGGAGAGGGAGGCCTAAGAAGCATTGGGGTGAGGTGGTTA
NbSOBIR1L-cDNA-P    ------------------------------------------------------------

NbSOBIR1-PS2        GACAGGACATGACCAGTATGCAGGTTACCGAGAACATGACACTTGATAGGGGAGTCTGGA
NbSOBIR1L-cDNA-P    ------------------------------------------------------------

NbSOBIR1-PS2        GGTCTAGAATCAGGATTGTAGGCTAGGGGCTTCTAGAGTCCGTCGTTCGCATCTCTCGGG
NbSOBIR1L-cDNA-P    ------------------------------------------------------------

NbSOBIR1-PS2        GTTGGGTGTGGGATATCTAGGGTTTCCCATAGGGGATTAGTGTTTTAGCTTATGTCTCCT
NbSOBIR1L-cDNA-P    ------------------------------------------------------------

NbSOBIR1-PS2        TATGAGTGTTAGTATGGTACTTACGGCTTGTTATTATTGCATTAATGCATGTGACTGTAT
NbSOBIR1L-cDNA-P    ------------------------------------------------------------

NbSOBIR1-PS2        TTATGTTGCTTATGCCTTCATACGTATTTACTTTATTTCTGCGGGAGTACCTATGCTTTA
NbSOBIR1L-cDNA-P    ------------------------------------------------------------

NbSOBIR1-PS2        TTATTTTGTTTTGTTTTGCTTGTGTTTCTAATTTCCCCTTTGTTTTCGTAATGCAGCTTT
NbSOBIR1L-cDNA-P    ------------------------------------------------------------

NbSOBIR1-PS2        TCCTCCCACATCCTATACGCCGGGGGTCGTTTGGAAGCAGCCTCTCTGCCCTTTCGGGCA
NbSOBIR1L-cDNA-P    ------------------------------------------------------------

NbSOBIR1-PS2        GGGGTAAGGCTCGTCTACCTATTACCCTCCCCAGACCCCACCTGGTGGGACTACCTGGGT
NbSOBIR1L-cDNA-P    ------------------------------------------------------------

NbSOBIR1-PS2        AGTTGTTGTTGTTGTTGTTGTTGTAGACGGATTGGCATCACTAGAAAAAAATTAGAAAAG
NbSOBIR1L-cDNA-P    -------------------------------------------------------AAAAG
                                                                           *****

NbSOBIR1-PS2        GTGGATGTGGAGAAGTTTATAAAGCAGAGTTACCAGGAAGTAATGGAAAGATTATTGCTA
NbSOBIR1L-cDNA-P    GTGGATGTGGAGAAGTTTATAAAGCAGAGTTACCAGGAAGTAATGGAAAGATTATTGCTA
                    ************************************************************

NbSOBIR1-PS2        TAAAGAAGATCGTAGAACCCCCGAAGGATGCTGCAGAACTCACTGAGGAAGATAGCAAGG
NbSOBIR1L-cDNA-P    TAAAGAAGATCGTAGAACCCCCGAAGGATGCTGCAGAACTCACTGAGGAAGATAGCAAGG
                    ************************************************************

NbSOBIR1-PS2        CTTTGAATATTAAAATGCGCCAGATTAAATCAGAAATTAAAATTGTAGGTCAAATAAGAC
NbSOBIR1L-cDNA-P    CTTTGAATATTAAAATGCGCCAGATTAAATCAGAAATTAAAATTGTAGGTCAAATAAGAC
                    ************************************************************

NbSOBIR1-PS2        ACACGAATTTGCTTCCTCTACTAGCACATATGCCAAGACCTGACTGCCATTACTTGATCT
NbSOBIR1L-cDNA-P    ACACGAATTTGCTTCCTCTACTAGCACATATGCCAAGACCTGACTGCCATTACTTGATCT
                    ************************************************************

NbSOBIR1-PS2        ACAAGTACATGAAAAATGGGAGCTTACAGGATGCCGTCCAGCAAGTCACGGAGGGGACAC
NbSOBIR1L-cDNA-P    ACAAGTACATGAAAAATGGGAGCTTACAGGATGCCGTCCAGCAAGTCACGGAGGGGACAC
                    ************************************************************

NbSOBIR1-PS2        GGGAAATAGATTGGTCGGTACGTCATCAAATTGCAGTCGGGATCGCTGCTGGGCTTGAGT
NbSOBIR1L-cDNA-P    GGGAAATAGATTGGTCGGTACGTCATCAAATTGCAGTCGGGATCGCTGCTGGGCTTGAGT
                    ************************************************************

NbSOBIR1-PS2        ATCTCCATGTAAATCATAGTCAGCGTATAATTCACAGGGATCTAAAGCCAGCAAATGTCC
NbSOBIR1L-cDNA-P    ATCTCCATGTAAATCATAGTCAGCGTATAATTCACAGGGATCTAAAGCCAGCAAATGTCC
                    ************************************************************

NbSOBIR1-PS2        TTCTTGATGATGACATGGAAGCTCGAATTACAGATTTTGGCTTTGCGAAAGCAATCCCAG
NbSOBIR1L-cDNA-P    TTCTTGATGATGACATGGAAGCTCGAATTACAGATTTTGGCTTTGCGAAAGCAATCCCAG
                    ************************************************************

NbSOBIR1-PS2        AATCCCTTACGCATGTTTCAACTTCACACGTGGTAGGAACTTTAGGATACATTGCACCAG
NbSOBIR1L-cDNA-P    AATCCCTTACGCATGTTTCAACTTCACACGTGGTAGGAACTTTAGGATACATTGCACCAG
                    ************************************************************

NbSOBIR1-PS2        AATATTACCAGACCGTTAAGTTCACAGATAAGTGTGATATATACAGATTCGGGGTGCTGC
NbSOBIR1L-cDNA-P    AATATTACCAGACCGTTAAGTTCACAGATAAGTGTGATATATACAGATTCGGGGTGCTGC
                    ************************************************************

NbSOBIR1-PS2        TAGGCGTGCTAGTTATGGGAAAGTTTCCCTCGGATGAGCACTTCCAGCCGGCTTCTGGGA
NbSOBIR1L-cDNA-P    TAGGCGTGCTAGTTATGGGAAAGTTTCCCTCGGATGAGCACTTCCAGCCGGCTTCTGGGA
                    ************************************************************

NbSOBIR1-PS2        TGGGTTTAGTGAAATGGATGAGAAATGTCATGACTTCTGAGAATCCAAAAAGAGCAATTG
NbSOBIR1L-cDNA-P    TGGGTTTAGTGAAATGGATGAGAAATGTCATGACTTCTGAGAATCCAAAAAGAGCAATTG
                    ************************************************************

NbSOBIR1-PS2        ATCCAAAGCTGATGGGTAATGGGTATGAGGAGCAAATGCTTTTGGTTCTCAAGATTGCCT
NbSOBIR1L-cDNA-P    ATCCAAAGCTGATGGGTAATGGGTATGAGGAGCAAATGCTTTTGGTTCTCAAGATTGCCT
                    ************************************************************

NbSOBIR1-PS2        GCTTTTGCAGCCTGGATAACCCAAAGGAGAGGCCACAATAA
NbSOBIR1L-cDNA-P    GCTTTTGCAGCCTGGATAACCCAAAGGAGAGGCCACAATAA
                    *****************************************
```

Supplementary Figure S6. Sequence alignment of predicted SOBIR1-like cDNA reported by Peng et al. (2015) with the corresponding sequence found in the *N. benthamiana* reference genome (NbSOBIR1-PS2). The aligned sequences extend from the start and stop codons of predicted cDNA. Predicted donor and acceptor splicing sites are underlined. Three nucleotide polymorphisms are located upstream of the first predicted donor splicing site, one of them being a single-nucleotide deletion.

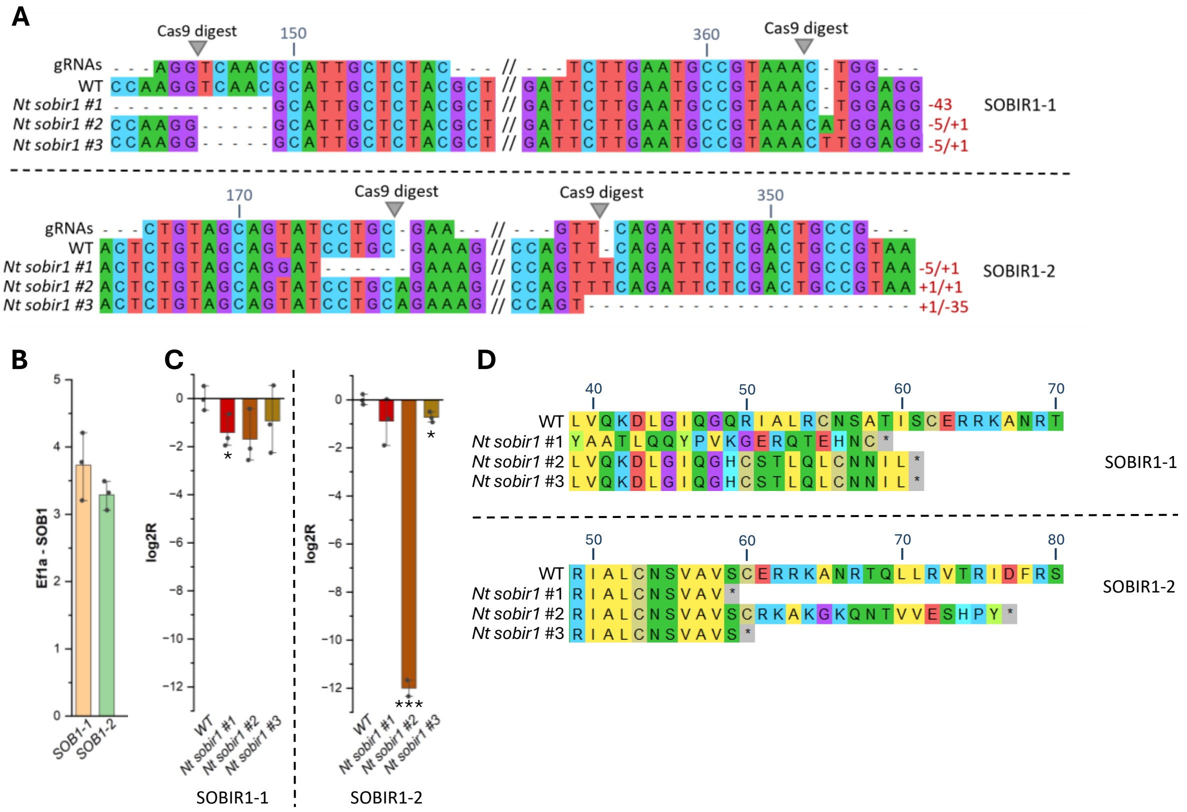

Supplementary Figure S7. Analysis of SOBIR1 DNA mutations, transcript levels, and predicted protein sequences in *Nt sobir1* mutant lines. (**A**) INDEL mutations in *SOBIR1* genes were identified in CRISPR/Cas9-edited *Nicotiana tabacum* lines. Targeted genomic regions were PCR-amplified and sequenced using the Sanger method. (**B**) Comparison of SOB1-1 and SOB1-2 expression in WT tobacco (**C**) Transcript levels of SOBIR1 in mutant lines were quantified by RT-qPCR. Amplified cDNA products were also verified by Sanger sequencing. (**D**) Predicted SOBIR1 protein sequences were inferred from genomic and transcriptomic data, highlighting the impact of CRISPR-induced mutations on protein structure.

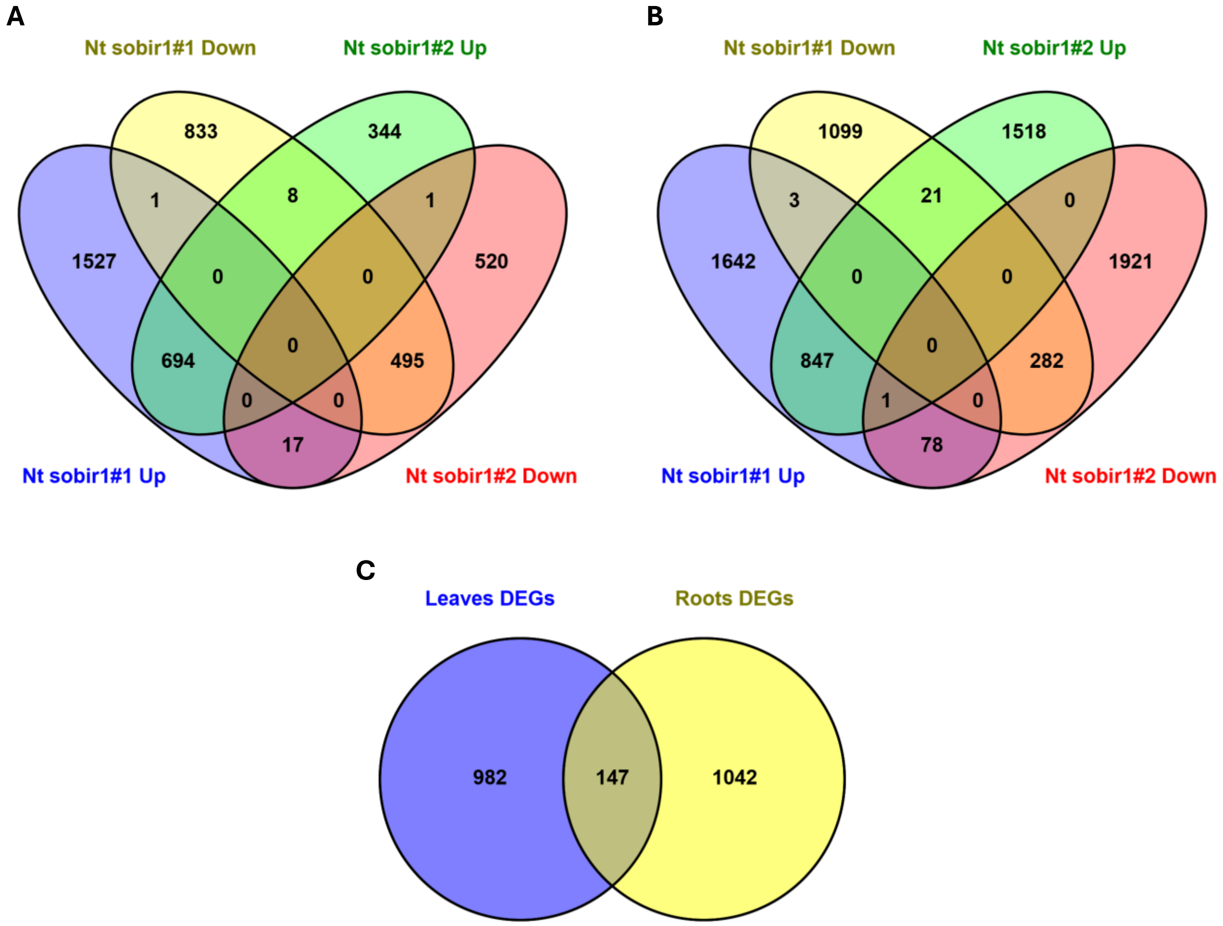

Supplementary Figure S8. **Tissue-specific and genotype-shared overlap of differentially expressed genes in *Nt sobir1* double mutant lines**. Overlap of differentially expressed genes (DEGs) identified in leaves (**A**) and roots (**B**) of the *Nt sobir1 #1* and *Nt sobir1 #2* double mutant lines. Panel (**C**) shows the intersection of DEGs that are commonly regulated in both double mutant lines across the two tissues.

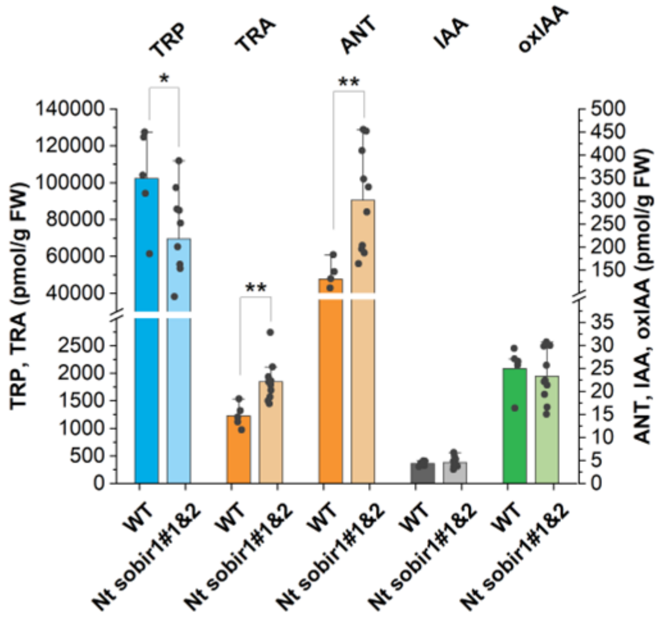

Supplementary Figure S9. Auxin profiling in *Nicotiana tabacum* wild-type (WT) and *Nt sobir1* double mutant lines. Quantification of indole-3-acetic acid (IAA), its biosynthetic precursors tryptophan (TRP), tryptamine (TRA), anthranilate (ANT), and the IAA degradation product 2-oxoindole-3-acetic acid. Metabolite levels were measured by LC-MS/MS and are presented as mean ± SD (n = 5). Asterisks indicate statistically significant differences relative to wild type (P < 0.05 (*), P < 0.01 (**); Student's t-test).

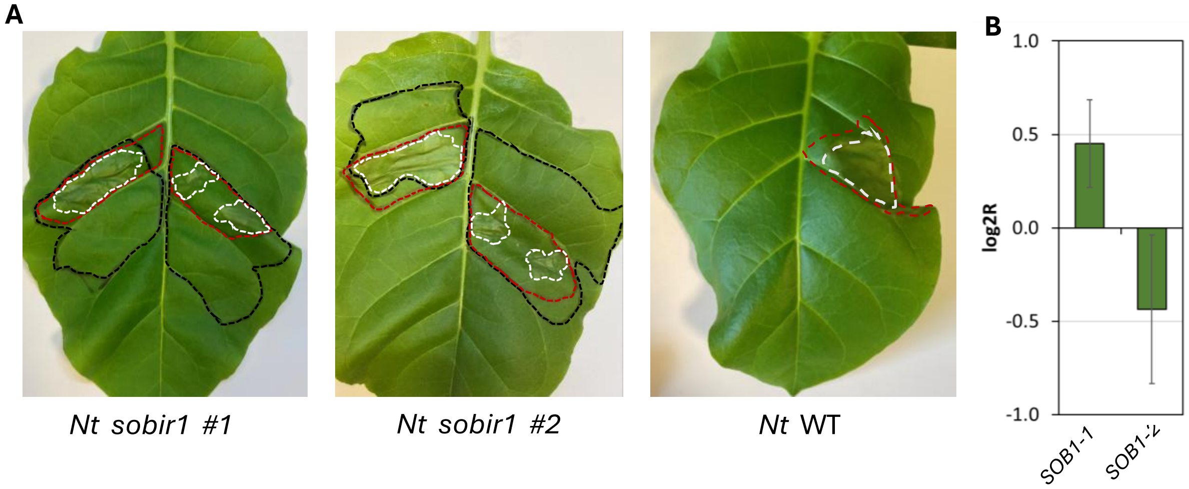

Supplementary Figure S10. Complementation of the *Nt sobir1* double mutants restores the elicitin-induced hypersensitive response (HR). (**A**) Transient expression of *NtSOBIR1-1* (left leaf part of the leaf) and *NtSOBIR1-2* (right part of the leaf) was performed via *Agrobacterium tumefaciens* infiltration (OD_{600} = 0.8) into leaves of *N. tabacum sobir1* double mutant plants (*Nt sobir1 #1* and *Nt sobir1 #2*). After 24 hours, 50 nM β-cryptogein was infiltrated into the same sites (red marks) and into the *N. tabacum* wild-type plant (*Nt* WT). The appearance of necrotic lesions (white marks) the following day indicated successful reactivation of the HR. (**B**) Transcript levels of transiently expressed *SOBIR1* were measured by RT-qPCR and normalized to endogenous *SOBIR1* expression in wild-type plants.

Complementation constructs containing premature stop codons were cloned into the pH7FWG2 binary vector under control of the CaMV 35S promoter. Experiments were independently repeated at least three times with consistent results.

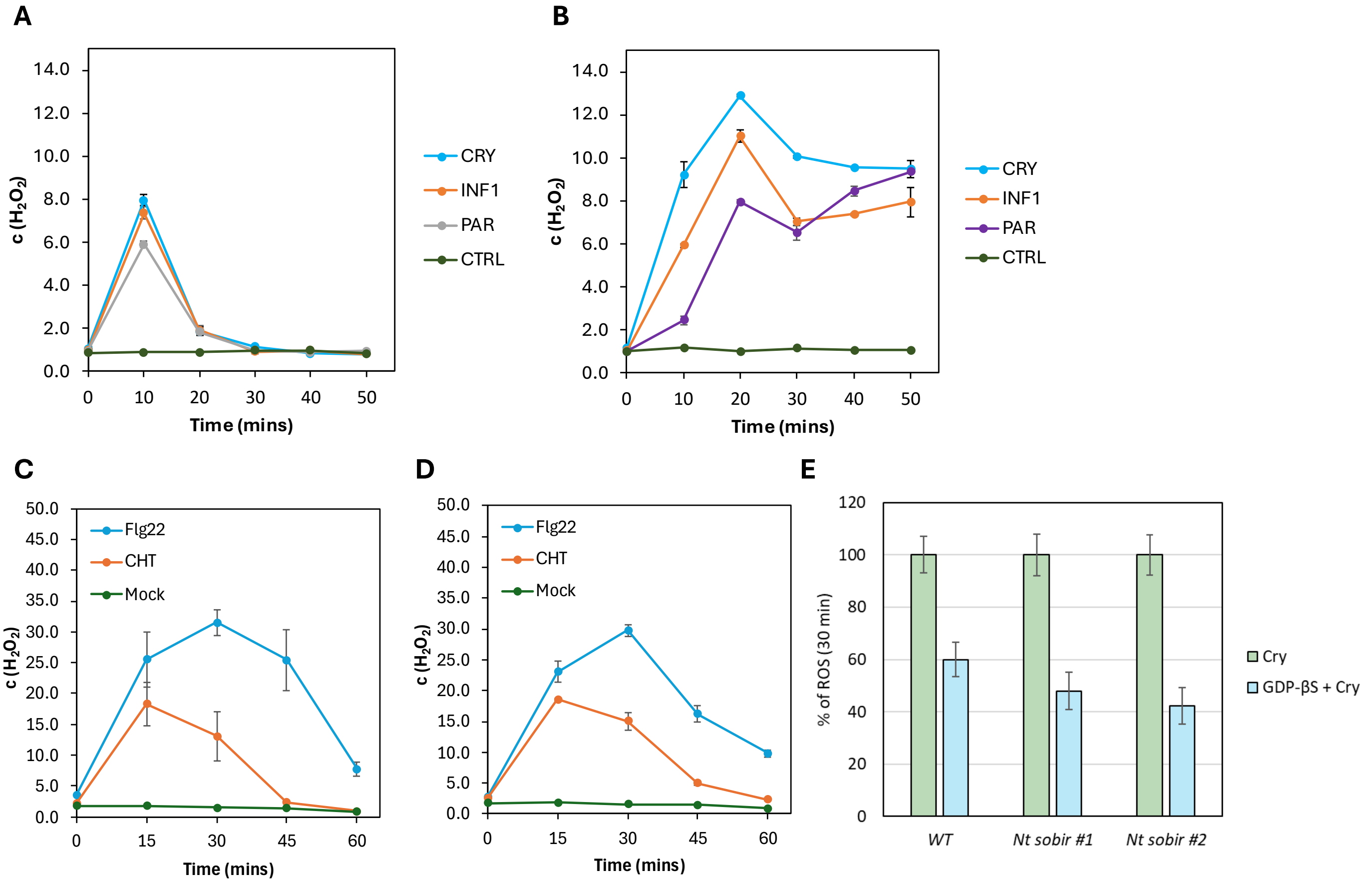

Supplementary Figure S11. Differential modulation of elicitor-induced ROS production by SOBIR1 in tobacco cell suspension cultures. (**A, B**) Reactive oxygen species (ROS, H_2O_2) production in 7-day-old *Nt sobir1 #1* double mutant (A) and wild type (WT) (B) suspension culture elicited with 50 nmol·L^{-1} CRY (β-cryptogein from *Phytophthora cryptogea*), 150 nmol·L^{-1} INF1 (infestin 1 from *Phytophthora infestans*) and 250 nmol·L^{-1} PAR (parasiticein from *Phytophthora parasitica*). Data represent means ± SD of two independent biological replicates (n = 2). (**C, D**) Reactive oxygen species (ROS; H_2O_2) production in 7-day-old *Nt sobir1 #1* double mutant (C) and wild type (D) suspension culture following elicitation with 1 µM flg22 (flagellin-derived peptide from *Xanthomonas* sp.) or 25 µg·L^{-1} chitosan from shrimp shells (Merck C3646, ≥75% deacetylated) (CHT). Data represent means ± SD of three independent biological replicates (n = 3). (**E**) Suspension cultures were pretreated with 500 µM GDP-βS for 10 min before stimulation with 100 nM β-cryptogein (Cry). Because GDP-βS exhibits limited stability in I_2 buffer (pH 7), ROS production was monitored for 30 min following elicitation. Data represent means ± SD of three independent biological replicates (n = 3). ROS accumulation was quantified using a luminol-based chemiluminescence assay.

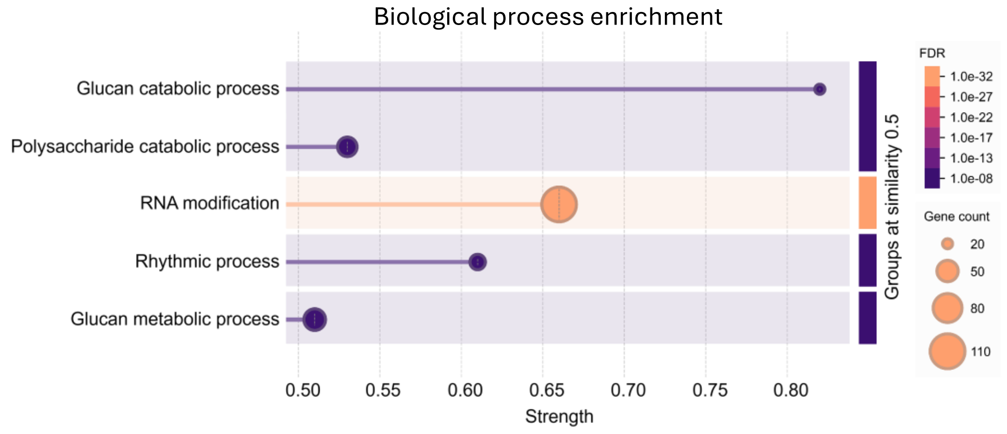

Supplementary Figure S12. Elicitin-induced transcriptomic changes in leaves of *Nicotiana tabacum sobir1*-double mutant lines (*Ntsobir1*) and wild-type (WT) plants. STRING Gene Ontology (GO) Biological Process enrichment dot plot of genes commonly downregulated (fold change > 2, Padj < 0.05) in both *Nt sobir1* #1 and #2 double mutants after β-CRY treatment. GO term descriptions are shown on the y-axis, enrichment strength on the x-axis, dot size indicates the observed gene count, and color represents the false discovery rate (FDR), as defined by STRING.

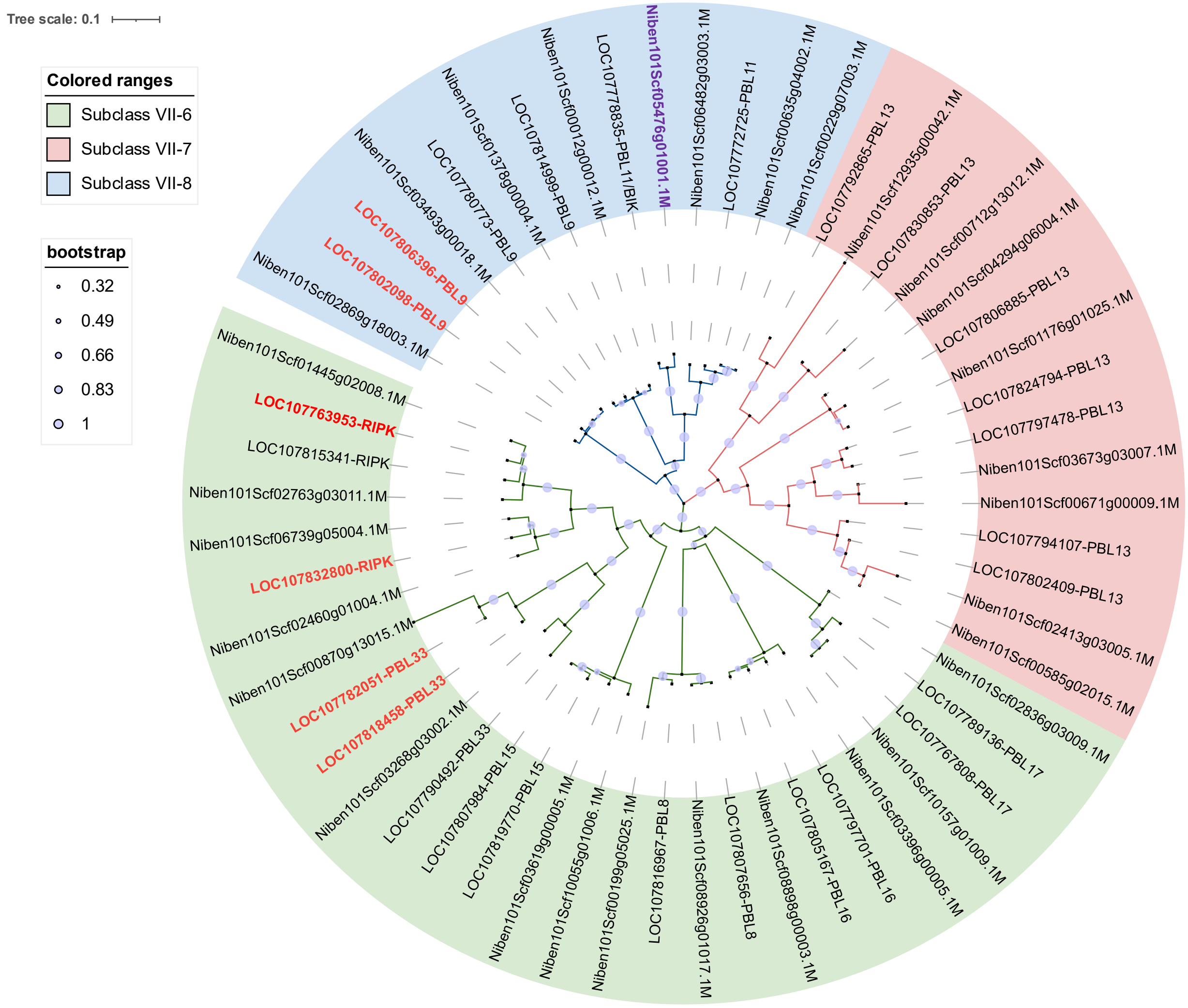

Supplementary Figure S13. Phylogenetic analysis of RLCK-VII-6, -7, and -8 subfamily members from *Nicotiana tabacum* and *Nicotiana benthamiana*. A total of 30 amino acid sequences previously identified as RLCK-VII-6, -7, and -8 subfamily members in *N. benthamiana* were used to identify 27 homologous sequences in *N. tabacum* via BLAST searches against the NCBI protein database. Phylogenetic analysis was conducted using the Neighbor-Joining method in MEGA11, with a bootstrap consensus tree generated from 500 replicates. All RLCK-VII members were assigned to subfamilies 6, 7, and 8 based on the classification system established for *N. tabacum* by Huang et al. (2024). Subfamilies are indicated in different colors. The putative *N. benthamiana* BIK1 homolog is highlighted in purple.

A

***Nt*SOBIR1-1**

5’ATGGCCTTCACTGCTTCACAAATCCACTTCTTTTTCTTCTCCCTTTTCGCCTTTTTACTTATTGCTGTTCAAGCAAGACTG
AATCTTTATCCACGAGATCATGCTGCACTTTTGCTTGTCCAAAAAGACCTGGGCATCCAAGGTCAACGCATTGCTCTACGCTG
CAACTCTGCAACAATATCCTGTGAAAGGCGAAAGGCAAACAGAACACAATTGTTGAGAGTCACCCGTATTGACTTCAGATCCA
ATGGATTGACTGGAACTTTATCTCCTGCCATTGGAAAACTTTCTGAGCTCAAAGAACTCTCTCTTCCAAACAACCAACTCTTT
GACCAAATCCCAGTTCAGATTCTTGAATGCCGTAAACTGGAGGTCCTTGACCTTGGAAACAATCTGTTTTCTGGGAAAGTTCC
ATCTGAATTATCATCTCTACTCCGCCTTCGAATTCTTGATCTTTCTTCAAATGAGTTGTCTGGGAATCTTAACTTCTTGAAGT
ATTT 3’

***Nt*SOBIR1-2**

5’ATGGCCTTCACTGCTTCACAAATCCACTTCTTTTTCATCTCCCTTTTCGCCCTTTTACTTATTGTTCAAGCAAAACTAAAT
CTTTATCCACCAGATCATGCTGCACTTTTGCTTGTCCAAAAAGACTTGGGCATCCAAGGTCAACGCATTGCACTTTGCAACTC
TGTAGCAGTATCCTGCGAAAGGCGAAAGGCAAACAGAACACAGTTGTTGAGAGTCACCCGTATTGACTTCAGATCCAATGGAT
TGAGTGGAACTTTGTCTCCTGCCATTGGCAAACTTTCTGAGCTCAAAGAGCTCTCTCTTCCAAACAACCGACTCTTTGACCAA
ATCCCAGTTCAGATTCTCGACTGCCGTAAACTGGAGATCCTTGACCTTGGAAACAATCTGTTTTCTGGGAAAGTTACATCTGA
ATTATCATCTCTAGTCCGCCTTCGAATTCTTGATCTTTCTTCAAATGAGTTGTCTGGGAATCTTAACTTCTTGAAGTATTTTC
CCAA 3’

B

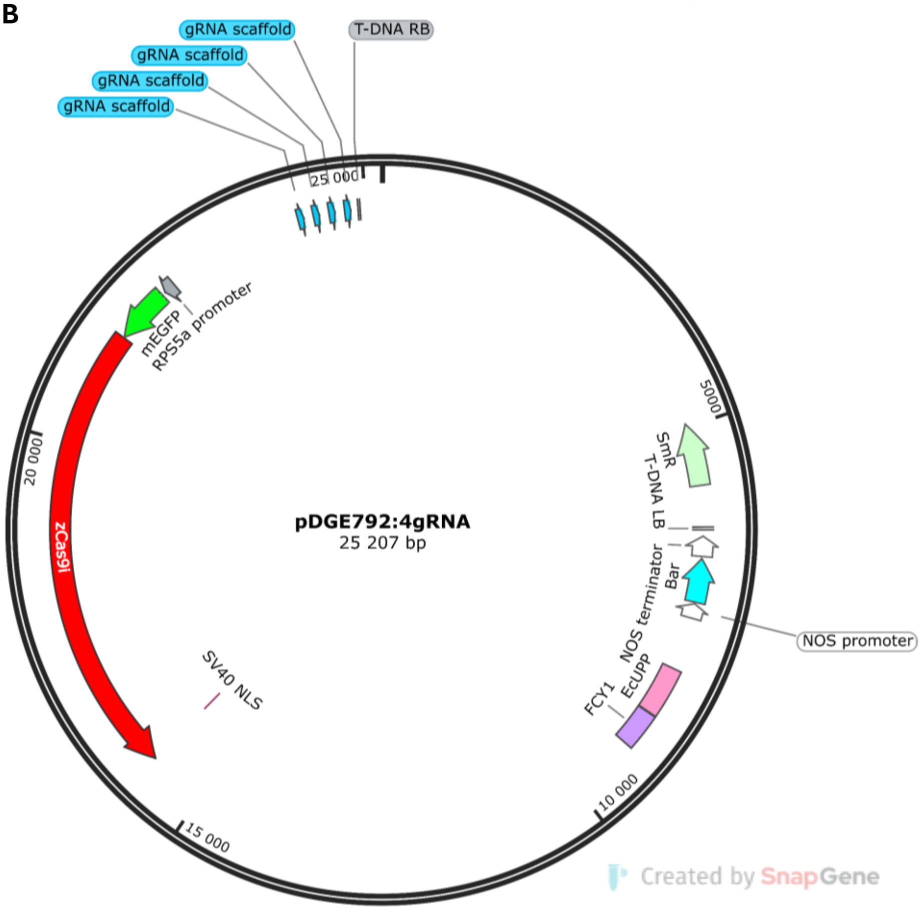

Supplementary Figure S14. Design of gRNA target sites in *NtSOBIR1* genes and structure of the CRISPR/Cas9 binary vector used for *Nicotiana tabacum* transformation. (**A**) Alignment of the first 500 bp of the *NtSOBIR1-1* and *NtSOBIR1-2* coding sequences, with Cas9-targeted gRNA protospacer sequences highlighted in red and PAM motifs (protospacer adjacent motifs) in blue. (B) Schematic representation of the CRISPR/Cas9 binary plasmid pDGE792:4gRNA. The construct encodes the intron-optimized Cas9 (zCas9i) fused to mEGFP under the control of the *RPS5A* promoter (Ribosomal Protein S5A). The zCas9i is further tagged with a SV40-derived nuclear localization signal (NLS) to ensure nuclear import. The plasmid harbors four gRNAs targeting both *NtSOBIR1* homologs. Additional selectable markers include SmR (spectinomycin resistance for bacterial selection), Bar (phosphinothricin resistance for plant selection), and FCY1 and EcUPP genes for negative selection in plants. The T-DNA borders (RB and LB) delimit the region transferred into plant cells during Agrobacterium-mediated transformation.
